# Mesoionic Carbene Complexes of Uranium(IV) and Thorium(IV)

**DOI:** 10.1021/acs.organomet.2c00120

**Published:** 2022-05-18

**Authors:** John A. Seed, Lisa Vondung, Ralph W. Adams, Ashley J. Wooles, Erli Lu, Stephen T. Liddle

**Affiliations:** Department of Chemistry, The University of Manchester, Oxford Road, Manchester M13 9PL, U.K.

## Abstract

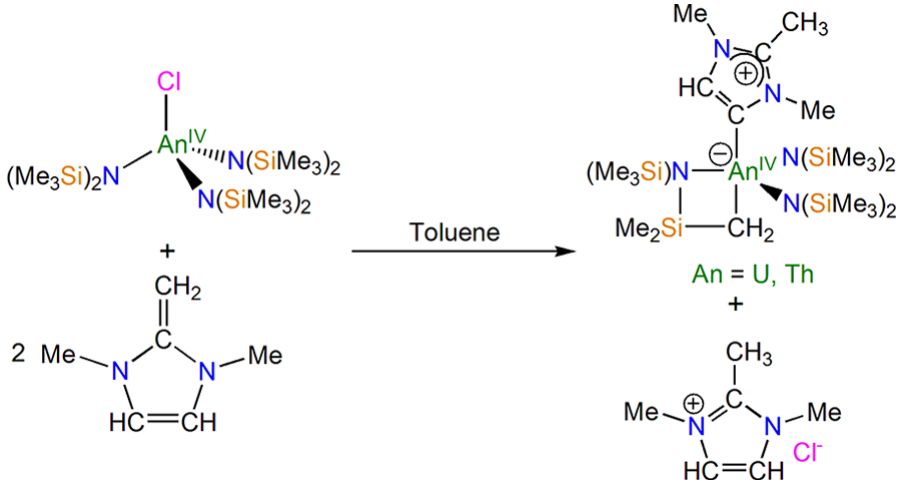

We report the synthesis and characterization of uranium(IV) and thorium(IV) mesoionic carbene complexes [An{N(SiMe_3_)_2_}_2_(CH_2_SiMe_2_NSiMe_3_){MIC}] (An = U, **4U** and Th, **4Th**; MIC = {CN(Me)C(Me)N(Me)CH}), which represent rare examples of actinide mesoionic carbene linkages and the first example of a thorium mesoionic carbene complex. Complexes **4U** and **4Th** were prepared via a C–H activation intramolecular cyclometallation reaction of actinide halides, with concomitant formal 1,4-proton migration of an *N*-heterocyclic olefin (NHO). Quantum chemical calculations suggest that the An–carbene bond comprises only a σ-component, in contrast to the uranium(III) analogue [U{N(SiMe_3_)_2_}_3_(MIC)] (**1**) where computational studies suggested that the 5f^3^ uranium(III) ion engages in a weak one-electron π-backbond to the MIC. This highlights the varying nature of actinide-MIC bonding as a function of actinide oxidation state. In solution, **4Th** exists in equilibrium with the Th(IV) metallacycle [Th{N(SiMe_3_)_2_}_2_(CH_2_SiMe_2_NSiMe_3_)] (**6Th**) and free NHO (**3**). The thermodynamic parameters of this equilibrium were probed using variable-temperature NMR spectroscopy yielding an entropically favored but enthalpically endothermic process with an overall reaction free energy of Δ*G*_298.15K_ = 0.89 kcal mol^–1^. Energy decomposition analysis (EDA-NOCV) of the actinide–carbon bonds in **4U** and **4Th** reveals that the former is enthalpically stronger and more covalent than the latter, which accounts for the respective stabilities of these two complexes.

## Introduction

Seminal work on carbenes by Bertrand and Arduengo increased the momentum of modern organometallic chemistry.^1^ The high stereoelectronic modularity of these versatile ligands has resulted in their wide use in a vast array of applications, while the elegant simplicity of their metal coordination has advanced the field of metal–*N*-heterocyclic carbene (NHC) complexes into a burgeoning area of research,^2^ the influence of which is reaching beyond that of the chemistry regime.^3^ Over the past three decades, a number of related stable carbenes have been developed, each with distinct steric and electronic properties,^4^ one example of which are mesoionic carbenes (MICs).^5^

MICs are dipolar heterocyclic stable carbenes whereby the free ligand is mesoionic, that is, no reasonable canonical resonance forms can be drawn without separated additional formal charges. Despite the progression of MIC chemistry in general, there are a comparatively limited number of examples reported, which mostly include free proligands or those which have formed in situ and remained within the primary coordination sphere of several transition-metal ions.^5^ In the bound state, a comparison with NHC ligands indicates MICs to be among the most electron-donating carbenes, with a significant part of their donor power derived from their zwitterionic character.^6^ Furthermore, Munz calculated the respective energies of the highest occupied molecular orbital (HOMO) carbene lone pairs and the lowest unoccupied molecular orbital (LUMO) π*-acceptor orbitals for different classes of carbenes, with MICs shown to be one of the strongest σ-donors, resulting in part from their high-energy HOMO, thus making the comparative underdevelopment of MICs somewhat surprising despite their limited synthetic routes.^6a^ This disparity is further emphasized within the f-block, where NHC f-element adducts, first isolated in 1994, are now well developed,^2h^ but MIC complexes remain exceedingly rare. Concerning actinide (An) derivatives, NHC complexes of uranium are well represented in the literature,^7^ but far fewer thorium–NHC complexes have been reported.^8^ This deficiency has been suggested to result in part from the increased lability of the weak Th–C_carbene_ linkage when coordinating the relatively soft NHC ligand to the hard thorium ion center but also likely has some basis in the lack of reliable synthetic methodologies to prepare these complexes analogously for both uranium and thorium. As such, there have been no examples of MICs of thorium, and apart from comprehensive reactivity studies,^8,9^ further developments in thorium cyclic carbene chemistry have been limited.

**Chart 1:**
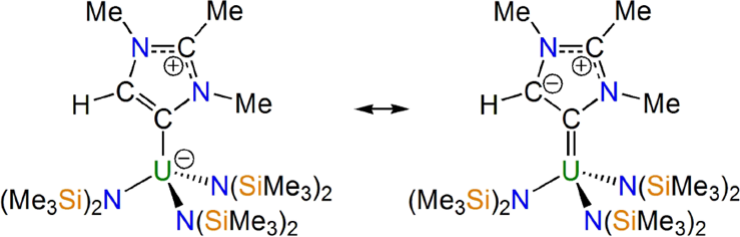
Two Principal Resonance Forms for the Previously Reported Uranium(III) MIC Complex **1**

Previously, we reported that employing *N*-heterocyclic olefins (NHOs) in reactions with trivalent f-element amides resulted in the isolation of f-block–MIC complexes including the first uranium–MIC complex, [U{N(SiMe_3_)_2_}_3_(MIC)] (**1**, MIC = {CN(Me)C(Me)N(Me)CH}). This synthetic methodology was found to be applicable to a number of trivalent rare-earth metals, highlighting a general method to develop complexes of this type.^10^ Quantum chemical calculations on **1** suggested that it exhibits a donor–acceptor character utilizing a single 5f electron from the uranium(III) ion in a weak π-backbond to the MIC in one of the two principal resonance forms for this complex (Chart 1). With a new synthetic pathway in hand, and the paucity of An–MIC complexes in general, tetravalent An–MIC derivatives were targeted in order to establish an understanding of how a change in the oxidation state of the 5f metal affects the electronic nature of the An–MIC interaction,^11^ in turn probing variations in An–MIC bonding as a function of oxidation state. Here, we report on the results of this endeavor, with the synthesis of two tetravalent An–MIC complexes along with the examination of the An–C bonding in these complexes.

## Results and Discussion

### Synthesis

**Scheme 1:**
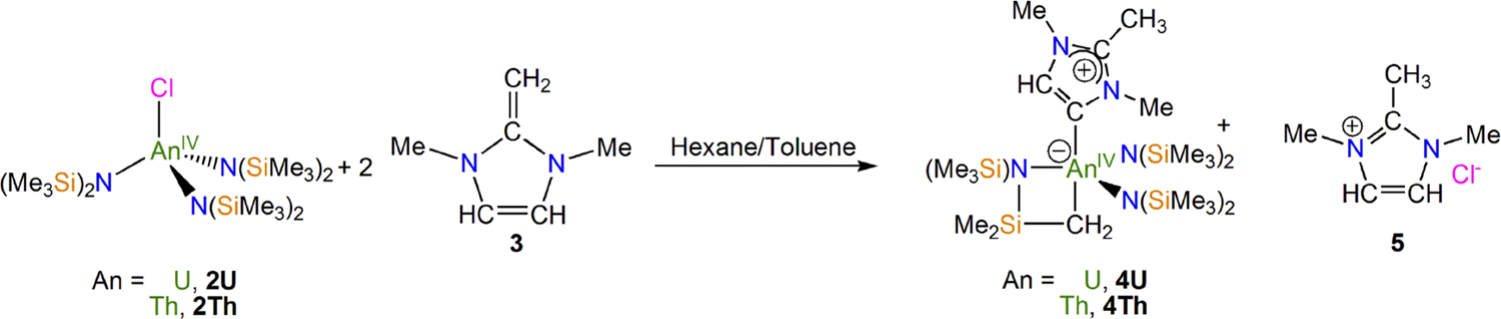
Synthesis of **4M**

Since *N*,*N*-bis(trimethylsilyl)amide, {(Me_3_Si)_2_N^–^}, had been successfully utilized as an ancillary ligand in the preparation of **1**, and to aid comparative purposes, we examined the utility of the An(IV)–triamide chloride complexes [An(Cl){N(SiMe_3_)_2_}_3_] (An = U, **2U** and An = Th, **2Th**) as precursors for the formation of An(IV) MIC complexes when reacted with the NHO [H_2_C=C(NMeCH)_2_] (**3**). Treatment of **2M** with **3** in toluene affords the cyclometalated An(IV) MIC, [An{N(SiMe_3_)_2_}_2_(CH_2_SiMe_2_NSiMe_3_)(MIC)] (An = U, **4U** and Th, **4Th**), instead of the anticipated An(IV)–triamide chloride MIC complexes [An{N(SiMe_3_)_2_}_3_(Cl)(MIC)], with the elimination of trimethylimidazolium chloride **5** observed as an insoluble side product (Scheme 1). Complexes **4M** represent the second and first examples of uranium and thorium MIC complexes, respectively, highlighting the applicability of this chemistry not only to other oxidation states of uranium but also to other early An metals.

**Scheme 2:**
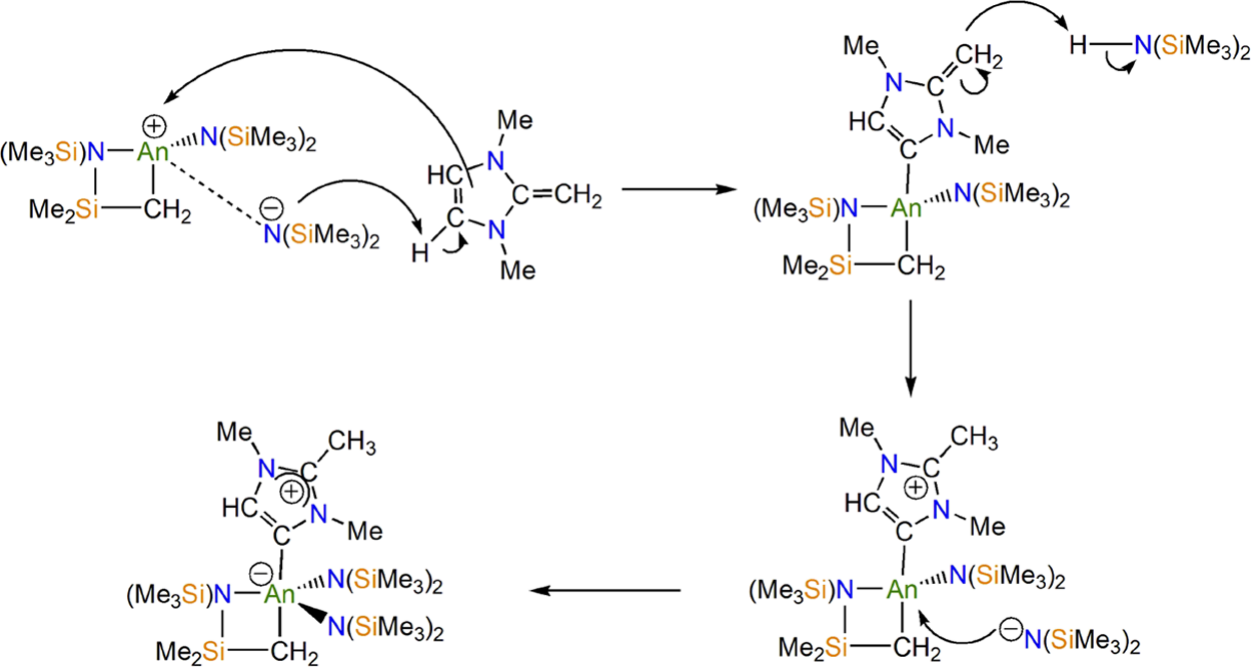
Proposed Mechanism for the Formation of **4M**

The formation of **4M** can be rationalized by a formal 1,4-proton migration of the NHO, an established pathway in accordance with the U(III) analogue, **1**, with coincident cyclometallation of one of the *N*,*N*-bis(trimethylsilyl)amide ligands.^10^ While the detailed mechanism is still unclear and the precipitation of insoluble **5** complicates any mechanistic reaction studies, it is reasonable to postulate that half an equivalent of the basic NHO, H_2_C^–^–C^+^(NRCH)_2_, **3**, deprotonates a SiMe_3_ group facilitating cyclometallation of the ligand framework with the elimination of trimethylimidazolium chloride **5** as a byproduct. The identity of **5** was confirmed by ^1^H nuclear magnetic resonance (NMR) spectroscopy in dimethyl sulfoxide-*d*_6_ (Figure S1 in the Supporting Information).^12^ Following this, C4-deprotonation of the other half equivalent of **3** could occur with the resultant NH(SiMe_3_)_2_ moiety reprotonating the putative An-intermediate, [An{N(SiMe_3_)_2_}(CH_2_SiMe_2_NSiMe_3_)(MIC)], at the basic methylene group to re-establish the An–amide bond, restore the overall charge neutrality to the MIC, and form **4M** (Scheme 2). In fact, the addition of a slight excess amount of **3** to [An{N(SiMe_3_)_2_}_2_(CH_2_SiMe_2_NSiMe_3_)] (**6M**) on an NMR scale results in the formation of **4M** suggesting that the cyclometallation of **2M** prior to rearrangement and subsequent coordination of **3** is a reasonable mechanistic suggestion (Figures S2 and S3 in the Supporting Information). We also note that when treating [Th{η^5^-C_5_H_3_(1,3-SiMe_3_)_2_}_3_], which is the cyclopentadienyl analogue of [U{N(SiMe_3_)_2_}_3_], that is, the direct precursor to **1**, no MIC formation is observed.^13^ However, it is currently not possible to discount a bimetallic mechanism or one where coordinated **3** is deprotonated by free **3** followed by isomerization and reprotonation.

### Solid-State Structures of **4M**

**Figure 1:**
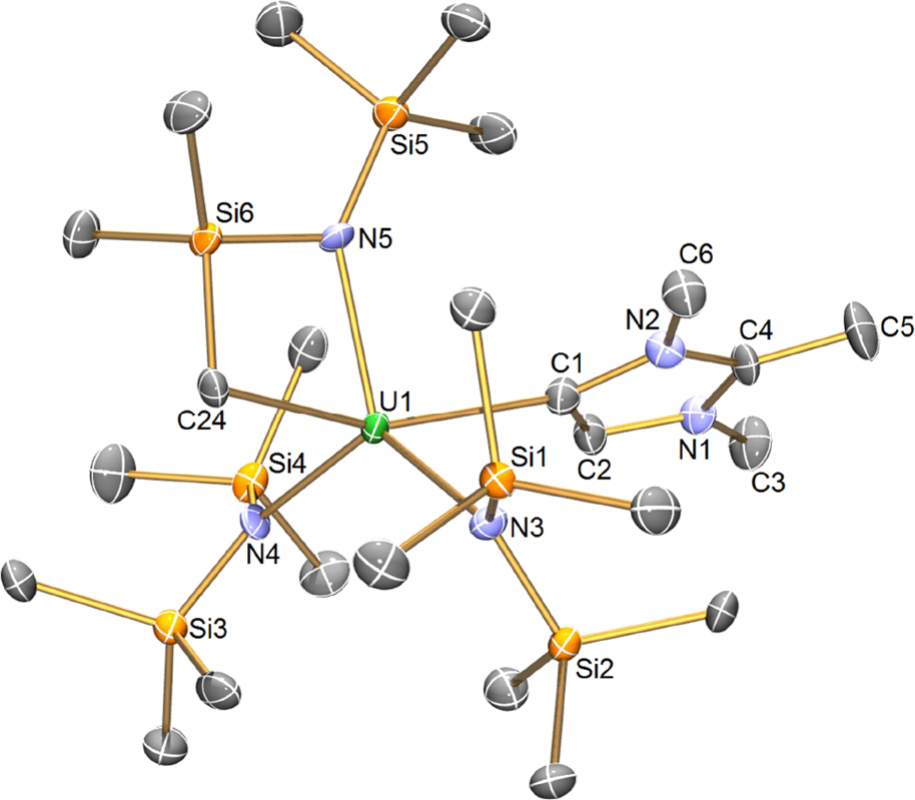
Molecular structure of **4U** at 150 K with displacement ellipsoids set to 30% probability. Hydrogen atoms and minor disordered components are omitted for clarity. The structure of **4Th** is very similar and is shown in the Supporting Information (Figure S4).

The molecular structures of **4M** were determined by single-crystal X-ray diffraction confirming their An–MIC formulations (Figures 1 and S4). In **4M**, the metal ions are five-coordinate and with τ values of 0.56 (**4Th**) and 0.53 (**4U**) adopting geometries that are essentially in between trigonal bipyramidal (τ = 1) and square-based pyramidal (τ = 0). For **4M**, the U–N_amide_ distances span a range of 2.280(7)–2.309(6) Å, while the Th–N_amide_ distances span a range of 2.342(2)–2.364(2) Å, suggesting the retention of the An +4 oxidation state.^7e,14,15^ The An–C_cyclomet_ distances of 2.460(9) Å in **4U** and 2.532(3) Å in **4Th** are within the range reported for uranium and thorium metallacycles generally (for U: 2.427(3)–2.545(6) Å and for Th: 2.449(12)–2.88(2) Å).^16^ For **4U**, the An–C_carbene_ distance of 2.618(10) Å is comparable to that of U(IV)–NHC complexes^7^ but is significantly longer than that found for U=C bonding interactions.^17^ While the An–C_carbene_ distance of 2.702(3) Å in **4Th** is comparable to the range observed for the thorium octa-NHC complex recently reported by the Jenkins and Arnold groups [2.6926(14)–2.7251(14) Å],^8a^ it is intermediate to that of other Th(IV)–NHC complexes [2.852(6)–2.884(5) and 2.623(6)–2.634(6) Å, respectively].^8b−8d^ Notably, the An–C_carbene_ distance in **4Th** is significantly longer than the range reported for Th=C bond interactions [2.2988(3)–2.489(14) Å].^15a,17f,18^ It is worth noting that while the U–C_carbene_ distance (in terms of the overall range by 3σ-criterion) of **4U** is longer than that of the reported U(III) MIC complex, **1**, [2.576(12) Å in **1** vs 2.618(10) Å in **4U**], the 3σ-overlap of these values and the differing coordination numbers of **4U** and **1** obviate any meaningful comparisons.

Considering **4M** together, the An–C_carbene_ distance is significantly longer in **4Th** [2.702(3) Å for **4Th** vs 2.576(12) Å for **4U**] as is the An–C_cyclomet_ distance [2.532(3) Å for **4Th** vs 2.460(9) Å for **4U**] compared to **4U**. While this difference is to be expected with the increased ionic radii of Th(IV) versus U(IV) (0.94 vs 0.89 Å, respectively),^19^ this is larger than anticipated and suggests the presence of a stronger and more developed An–C interaction in **4U** versus that of **4Th**.

### Magnetic Properties of **4U**

**Figure 2:**
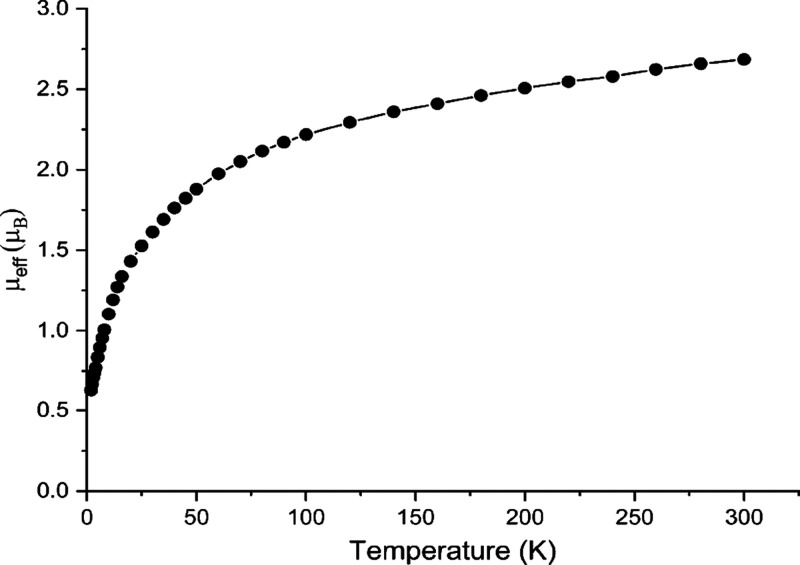
Temperature-dependent SQUID data for powdered samples of **4U** recorded in a 0.1 T magnetic field over a temperature range of 2 to 300 K. The line is a guide to the eye only.

A powdered sample of **4U** was measured by variable-temperature SQUID magnetometry in an applied external field of 0.1 T (Figures 2 and S5 in the Supporting Information). The magnetic moment of **4U** at 300 K is 2.68 μ_B_, and this value decreases smoothly over the temperature range reaching 0.63 μ_B_ at 2 K. This magnetic behavior is characteristic of a ^3^H_4_ uranium(IV) ion, which is a magnetic triplet at room temperature and a magnetic singlet at low temperature subject to temperature-independent paramagnetism.^20,21^

### Ultraviolet–visible–Near-Infrared (UV–vis–NIR) Spectroscopy of **4U**

For **4U**, the UV/vis/NIR spectrum is dominated by metal-to-ligand charge transfer, which occurs at high energy (<500 nm), while multiple weak formally Laporte-forbidden f–f transitions can be observed in the NIR region of the spectrum, characteristic of a ^3^H_4_ uranium(IV) ion (Figures S6 and S7 in the Supporting Information).^11^

### NMR Spectroscopy of **4U** and **4Th**

The expected 36:9:6:2 ratio for a bis(trimethylsilyl)amide metallacycle is observed upon inspection of the ^1^H NMR spectra for **4M** along with all expected resonances of the MIC ligand. In the case of **4U**, the resonances span a wide range with the uranium-bonded CH_2_ group being the most shielded resonating as a singlet at −119.84 ppm, a consequence of the direct interaction with the paramagnetic U(IV) center. Furthermore, the heterocyclic C–H of the MIC ligand in **4U** resonates as a singlet at −18.53 ppm in the ^1^H NMR spectrum, shielded in a comparable way to the heterocyclic C–H in the U(III) analogue **1**. Overall, the ^1^H NMR spectrum of **4U** is consistent with its An(IV) formulation, with the respective ^1^H NMR spectrum being remarkably similar to that reported for the MIC-free U(IV) metallacycle **6U**,^15d^ suggesting that coordination of the MIC has little effect on the electronic nature of the U(IV) center (Figures S8 and S9 in the Supporting Information). The paramagnetic nature of the U(IV) ion in **4U** precluded any meaningful assignment of ^29^Si{^1^H} spectra due to line broadening.

**Scheme 3:**
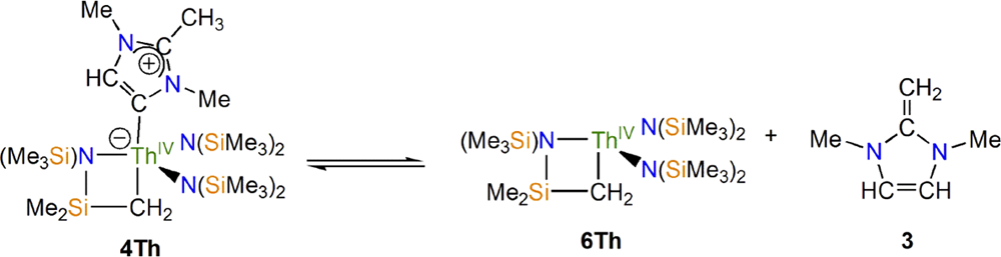
Solution Equilibrium of **4Th** with **6Th** and **3**

In the diamagnetic thorium analogue, **4Th**, a much smaller spectral range of 6.50–0.30 ppm is observed with the thorium-bonded CH_2_ group resonating as a singlet at 0.66 ppm in the ^1^H NMR spectrum, while the heterocyclic C–H resonance is observed as a singlet at 6.48 ppm. The ^29^Si{^1^H} NMR spectrum of **4Th** exhibits the expected resonances assigned to the three silicon environments at −10.58, −11.32, and −23.96 ppm, respectively (Figures S10 and S11 in the Supporting Information). However, for **4Th**, the ^1^H and ^29^Si{^1^H} NMR spectra show that in a C_6_D_6_ solution, **4Th** exists in equilibrium with **3** and the MIC-free Th(IV) metallacycle, **6Th**, Scheme 3, with the resonances attributed to **6Th** shifted relative to that of a pure sample (Figures S12–S16 in the Supporting Information).^15d^ At 298 K, the equilibrium favors **6Th** and **3** so **4Th** has a low concentration which, along with slow ^13^C longitudinal relaxation, hampered attempts to record its ^13^C NMR spectrum since all that could be observed are **6Th** and **3**. Nevertheless, using a heteronuclear multiple bond correlation (HMBC) measurement to overcome the low sensitivity and slow relaxation that hamper direct ^13^C acquisition, we were able to ascertain the ^13^C chemical shifts of **4Th**. The HMBC (Figure S17 in the Supporting Information) reveals that the cyclometallate carbon resonates at 6.4 ppm and the MIC carbene center resonates at 208.4 ppm, which for the latter is similar to the carbene chemical shifts of thorium–NHC complexes.^8^ HMBC also allowed detection of the **4Th**^13^C_α_ (C=CH) signal at 128.9 ppm, which overlaps with the C_6_D_6_ solvent signal at 128.1 ppm.

The addition of excess **3** does not alter this equilibrium noticeably; hence, this solution behavior is suggested to be a result of a weak Th–MIC interaction in **4Th** causing partial dissociation of the MIC moiety in solution, further emphasized by the lack of an analogous solution behavior by **4U** that is consistent with the structural and computational analyses (vide infra) of the presence of a more developed An–MIC interaction in **4U** versus that of **4Th**.

The equilibrium behavior for the interconversion of **4Th** into **6Th** and **3** in toluene-*d*_8_ was probed through variable-temperature NMR spectroscopy experiments over a temperature range of 298.15–253.15 K (Figures S18 and S19 in the Supporting Information). The van’t Hoff plot determined using the equilibrium concentrations is linear (*R*^2^ = 0.9931, Figure S20 in the Supporting Information) and reveals an entropically favored but enthalpically endothermic process (Δ*H* = 4.69 kcal mol^–1^ and Δ*S* = 12.77 e.u.) with a reaction free energy of Δ*G*_298.15K_ = 0.89 kcal mol^–1^. That is, as the thermal energy of the system is increased with temperature, so does the rate of conversion of **4Th** into **6Th** and **3** increases. The thermochemistry of the phosphorano-stabilized thorium carbene [Th(CHPPh_3_){N(SiMe_3_)_2_}_3_] (**7**) was investigated by Hayton and co-workers.^15a^ A comparison with that of **4Th** suggests that while the conversion of both carbenes into their respective constituents follows an entropically driven process, the enthalpic component is more positive in **7** than in **4Th** (Δ*H* = 9.4 kcal mol^–1^ for **7** vs Δ*H* = 4.69 kcal mol^–1^ for **4Th**) suggestive of a more thermodynamically favorable conversion of **4Th** into **6Th** and **3** than **7** into **6Th** and CH_2_ = PPh_3_. The difference in enthalpy is to be expected, however, when the metal–carbene bonds being formed and broken in each case are considered. For **7**, the Th–C interaction is best described as consisting of a two-center two-electron σ-bond with a three-center two-electron Th–C–P π-component. In contrast, the Th–MIC interaction in **4Th** is solely a dative-type σ-bond with no π-component present (vide infra), resulting in lower activation parameters for the bond breakage in the latter than the former.

### Computational Analysis of **4U** and **4Th**

In order to provide insight into the electronic structures of **4M** and to rationalize the NMR and thermodynamic data, a density functional theory (DFT) analysis was performed to gain more insight into the nature of the An–MIC bond. The MIC–U two-electron σ-donation interaction in **4U** is represented by HOMO–16, whereas the C_cyclomet_–U two-electron σ-donation interaction is represented by HOMO–2 (Figure S21 in the Supporting Information). There is no U–MIC π-backdonation present with HOMO and HOMO–1, which accounts for the two, nonbonding 5f electrons of the U(IV) oxidation state. For **4Th**, the MIC–Th two-electron σ-donation interaction is represented by HOMO–14, whereas the C_cyclomet_–Th two-electron σ-donation interaction is represented by the HOMO, consistent with the closed shell configuration of the d^0^f ^0^ Th(IV) ion (Figure S22 in the Supporting Information). The calculated Nalewajski–Mrozek bond orders around the MIC rings of **4U** and **4Th** span a narrow range of 1.10–1.36, except for the formal C=C bond which is 1.69 in **4U** and 1.71 in **4Th**, suggesting that the change in An metal, from uranium to thorium, has little effect on the electronic structure of the MIC ring. The uranium–carbene bond order is calculated to be 0.72, which is larger than the thorium–carbene bond order of 0.50 but both are significantly lower than that of **1** (1.10). The uranium–cyclometalate bond order is calculated to be 1.13, whereas the thorium–cyclometalate bond order is calculated to be 0.82. These comparatively high bond orders perhaps reflect that the cyclometalate is a formally anionic donor, whereas the MIC is a neutral, dative donor overall. In accordance with the long U–C_carbene_ bond distance in **4U**, the high bond order of U–C_cyclomet_ suggests that a *trans* influence is in operation, a phenomenon well accepted throughout the transition-metal chemistry and now being reported for mid-valent uranium complexes even when the interligand bond angles deviate from 180°.^17h^

For **4U**, the MIC–U σ-donation is determined by natural bond orbital (NBO) analysis but is returned as essentially electrostatic and so this orbital is predominantly carbon-based with the carbene acceptor orbital being empty with no 5f ^2^ contribution (Figure S23 in the Supporting Information). For **4Th**, the NBO analysis returns all the ligand lone pairs as ligand-localized and so no meaningful insight can be gained from this analysis.

To further understand the nature of the metal–carbene linkages in **4M** in addition to the orbital-based perspectives provided by DFT and NBO analyses, we probed the topological electron density description of these An–C_carbene_ bonds. For **4U**, the ρ(*r*)_UC_ value of 0.05 suggests a rather polar interaction since covalent bonds tend to have ρ(*r*) > 0.2. The calculated ellipticity parameter ε(*r*)_UC_ of 0.06 suggests a cylindrical σ-bond between uranium and carbene in **4U** with no π-bonding component involved in agreement with the spectroscopic data.^22^ For **4Th**, a ρ(*r*)_ThC_ value of 0.04 and a calculated ellipticity parameter ε(*r*)_ThC_ of 0.01 also suggest a cylindrical σ-bond with no π-bonding component involved. This is in stark contrast with **1**, where an ε(*r*)_UC_ value of 0.36 was computed for the U=C_carbene_ bond. For both **4M**, the ρ(*r*)_MCcyclomet_ values of 0.08 suggest a rather polar interaction, though more covalent than the An–C_carbene_ bonds in-line with the data discussed above. Again, the calculated ellipticity parameter ε(*r*)_MC_ of 0.10, although deviating from zero presumably due to its skewed binding C–M–C angle [157.5(3) and 153.74(10)° for **4U** and **4Th**, respectively] from the constraints of the cyclometalate four-membered ring, is consistent with the σ-bond between the metal center and C_cyclomet_.

With MIC complexes in hand across different An metals in addition to differing oxidation states, it is instructive to compare the Nalewajski–Mrozek bond orders of the respective U(III), **1**, U(IV), **4U**, Th(IV), and **4Th** MICs (Table 1).

**Table 1:** Comparison of Nalewajski–Mrozek Bond Orders for **1**, **4U**, and **4Th**

	Nalewajski–Mrozek bond orders		
bonding component	**1**	**4U**	**4Th**
C–N	1.22	1.26	1.27
C=C	1.64	1.69	1.71
M–C_carbene_	1.1	0.72	0.5
M–C_cyclomet_		1.13	0.82

The M–C_carbene_ bond order of **1** is significantly larger than those of both **4U** and **4Th** with **4Th** possessing the lowest M–C_carbene_ bond order of the three. A bond order of less than one for **4M** is suggestive of solely a σ-component to their bonding and an electrostatic An–carbene interaction. For **4U**, the energy differences between the 5f orbitals and the carbene frontier orbitals would appear to be large enough to prevent any orbital interaction, that is, covalent backdonation, and therefore the higher M–C bond order in comparison to **4Th** arises due to a more strongly developed σ-bond (HOMO–16). A weaker M–C_carbene_ interaction should result in the strengthening of the C_carbene_–α–C and C_carbene_–α–N bonds, respectively, and this can be visualized by the increasing C_carbene_–α–C and C_carbene_–α–N bond orders from U(III) to U(IV) to Th(IV) (1.64/1.22, 1.69/1.26, and 1.71/1.27, respectively). In addition to this, **4U** exhibits a significantly higher M–C_cyclomet_ bond order compared to **4Th**. This lower bond order of **4Th** can be attributed to the lengthening of the M–C_cyclomet_ bond distance as a result of the increased ionic radii of Th(IV) in comparison to U(IV) (0.94 vs 0.89 Å).^19^ This lengthening could manifest in a poorer p-orbital overlap and a significant reduction in the bond order. Despite this, the bond orders within the MIC ring are similar for both **4M**, suggesting a little difference in the electronic structure of the MIC ring.

In terms of bonding symmetry, while **1** contains both σ- and π-components, to the M=C_carbene_ bonding interaction, **4M** only contain the σ-component with the carbene acceptor orbital being formally empty. Additionally, the U=C_carbene_ bond in **1** exhibits a significant degree of covalency for the π-component, while both the Th–C_carbene_ bond in **4Th** and the U–C_carbene_ bond in **4U** are largely ionic. The f ^3^ nature of **1** facilitates a singly occupied 5f orbital, HOMO–1, energetically compatible with the frontier orbitals of C_carbene_ thus enabling it to participate in U–MIC π-backbonding. In contrast, the closed shell f ^0^d^0^ configuration of **4Th** precludes metal–carbene π-backdonation, while the 5f ^2^**4U** possesses two nonbonding 5f electrons. Hence, while **4Th** does not possess the requisite electrons to participate in metal–carbene π-backdonation, **4U** does, but they are energetically incompatible to do so; therefore, only σ-bonding occurs.

**Figure 3:**
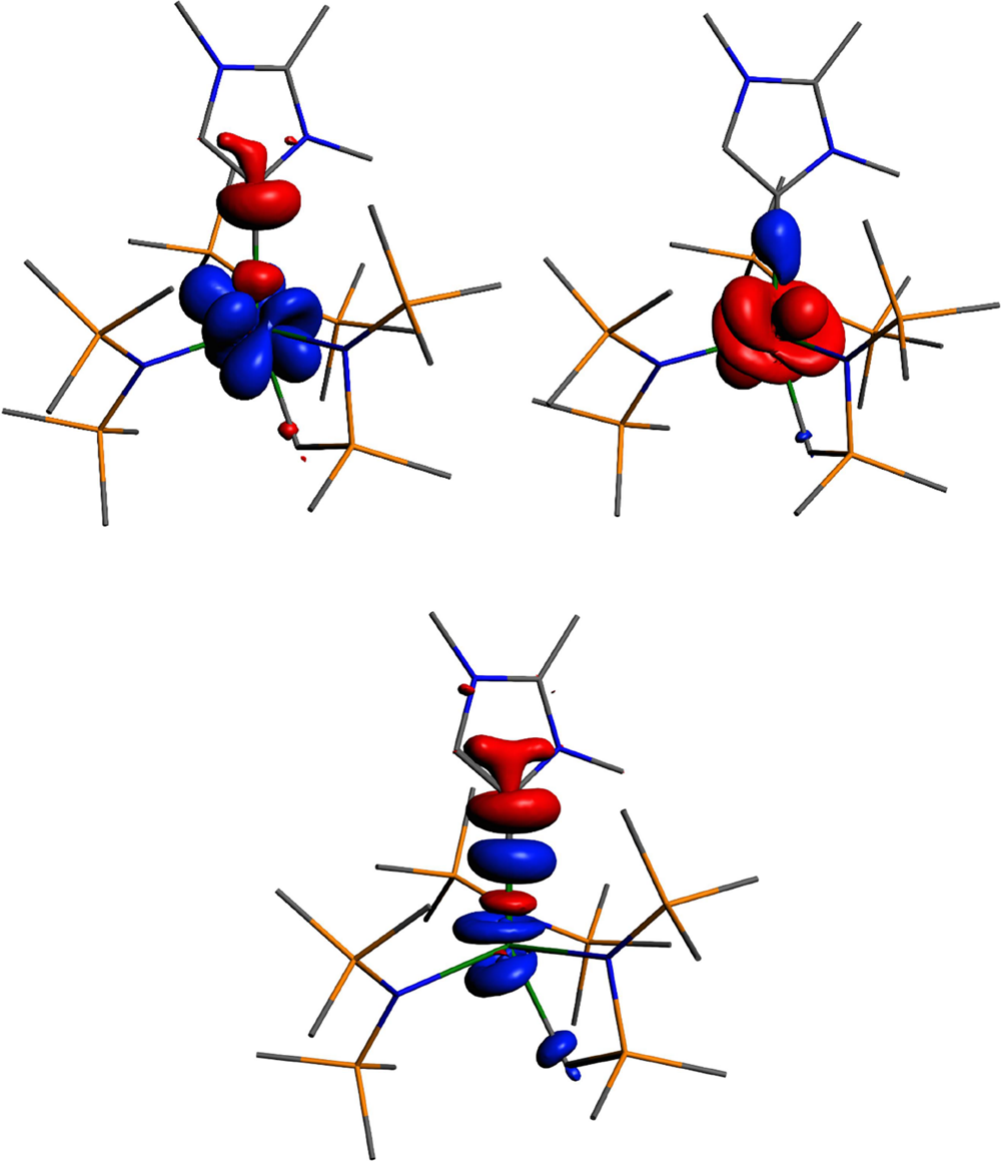
Top: Deformation densities Δρ_(1α)_ for α (left) and Δρ_(1ß)_ for ß (right) spins with the highest contribution to Δ*E*_orb_ in **4U**, Δ*E*_1α_ = −41.40 kcal/mol, |ν_1α_| = 0.28 and Δ*E*_1ß_ = 32.62 kcal/mol, |ν_1ß_| = 1.00. Bottom: Deformation density for **4Th**. Δρ_(1)_, Δ*E*_1_ = −19.6 kcal/mol and |ν_1_| = 0.45. The charge flow is red → blue. H-atoms are omitted for clarity.

Additional insights into the An–MIC bond were obtained from an energy decomposition analysis in combination with natural orbitals for chemical valence (EDA-NOCV). This method allows partitioning of the bonding interaction between the neutral MIC and [An{N(SiMe_3_)_2_}_2_(CH_2_SiMe_2_NSiMe_3_)] fragments into Coulomb (Δ*E*_elstat_), orbital (Δ*E*_orb_), and Pauli (Δ*E*_Pauli_) contributions (Table S1 in the Supporting Information). The deformation densities, Δρ, associated with the various contributions to Δ*E*_orb_ can then be visualized to exhibit the charge flow during bond formation. The total An–MIC bond interaction energies show that the U–MIC bond is stronger than the Th–MIC bond (−39.0 kcal/mol for **4U** vs −29.9 kcal/mol for **4Th**), consistent with the higher An–MIC bond order in **4U** (0.72) in comparison with that of **4Th** (0.50). Examining the respective bonding contributions in **4U** and **4Th** reveals two components, both a Coulombic (electrostatic) attraction and a covalent (orbital) attraction to the bonding (62:38% for **4U** and 69:31% for **4Th**), in agreement with the predominantly ionic character of the An–MIC bonds highlighted in the aforementioned QTAIM metrics and also more covalency in the U–MIC bond than in the Th–MIC bond. The plotted deformation densities from the pairwise interaction of the NOCVs with the highest contributions to Δ*E*_orb_ show a similar interaction for both **4U** and **4Th** (Figures 3 and S24 and S25 in the Supporting Information).

In **4Th**, only one deformation density contributes to Δ*E*_orb_ above the cutoff value of 5 kcal/mol. In this case, electron density is donated from carbene to the thorium metal center, resulting in solely a σ-type bonding, in agreement with the QTAIM analysis. For **4U**, the nature of unrestricted calculations means that the deformation densities are split into α and ß densities, making the interpretation less straightforward. However, taking both densities together, it becomes clear that as in the case of **4Th**, a σ-bond is formed by overall donation from carbene to the uranium metal center. For **4U**, further deformation densities of lower contribution to Δ*E*_orb_ were found (Figures S24 and S25 in the Supporting Information), which show small additional donation from the carbene backbone as well as a very small contribution to potential π-backbonding from uranium to carbene, indicating a more varied and covalent bonding picture in **4U** compared to that of **4Th**.

The observed difference in the solution behavior of **4U** and **4Th** can thus be rationalized by the computed An–MIC bond strengths and the bond dissociation energies. The total bonding interaction between the actinide fragment and the MIC ligand is found to be stronger for **4U** (−39 kcal/mol) than for **4Th** (−30 kcal/mol). The bond dissociation energies of −*D*_e_ = −29.5 kcal/mol for **4U** and −22.5 kcal/mol for **4Th** also explain the observed higher stability of **4U** in solution and the divergent equilibrium behavior of **4Th**. Interestingly, this trend is in contrast to that observed by Hayton and co-workers in **7** and the corresponding uranium analogue, [U(CHPPh_3_){N(SiMe_3_)_2_}_3_],^14b^ but a direct comparison of these systems is difficult as in the formation of **4M** where only one An–C bond is broken, whereas Hayton’s system undergoes one An–C bond breakage and one An–C bond formation.

## Conclusions

To conclude, we have prepared rare examples of An–MIC complexes, [An{N(SiMe_3_)_2_}_2_(CH_2_SiMe_2_NSiMe_3_)(MIC)] where An = U or Th (**4U/Th**), including the first example of an MIC complex of thorium. The similarities between the two analogous tetravalent carbene complexes, **4U** and **4Th**, which is not only limited to their equivalent mechanism of formation and structure but also the electronic nature of the metal–MIC interaction, highlight the applicability of this chemistry across the 5f series. Despite this, it is clear that subtle electronic and structural changes can result in significant differences in the nature of An–MIC bonding. As such, it is notable that for the U(III) system, **1**, both σ- and π-bonding are in operation, whereas for the U(IV) system, **4U**, only σ-bonding is exhibited. Additionally, the An–MIC bond is slightly more covalent and stronger in **4U** than in **4Th**, resulting in a more stable solution behavior for **4U** with partial dissociation in solution observed for **4Th**. Further extrapolation of this MIC chemistry across the 5f series to transuranic elements would provide an ideal platform to investigate how the change in 6d/5f energies, and the increasing number of valence electrons, alters the nature of the metal–MIC interaction across the An series.

## Methods

### General Experimental Details

All manipulations were carried out using Schlenk techniques or an MBraun UNIlab glovebox under an atmosphere of dry nitrogen or argon. Solvents were dried by passage of activated alumina towers and degassed before use. All solvents were subsequently further dried and stored over NaK_2_. Deuterated solvents were dried over NaK_2_, distilled, and stored over NaK_2_. Glassware used for all novel reactions was silylated with HMDS under a reduced pressure. Crystals (see Table S2) were examined using an Agilent SuperNova diffractometer equipped with an Eos CCD area detector and a Microfocus source with Mo Kα (λ = 0.71073 Å) and Cu Kα (λ = 1.54184 Å) radiation for **4Th** and **4U**, respectively. Intensities were integrated from data recorded on narrow (0.5°) frames by ω rotation. Cell parameters were refined from the observed positions of all strong reflections in each data set. Gaussian grid face-indexed absorption corrections with a beam profile correction were applied. The structures were solved by direct methods, and all nonhydrogen atoms were refined by the full-matrix least-squares method for all unique *F*^2^ values with anisotropic displacement parameters with exceptions noted in the respective cif files. Except where noted, hydrogen atoms were refined with constrained geometries and riding thermal parameters. CrysAlisPro^23^ was used for control and integration, SHELXT^24^ was used for structure solution, and SHELXL^25^ and Olex2^26^ were employed for structure refinement. ORTEP-3^27^ and POV-Ray^28^ were employed for molecular graphics. ^1^H, ^13^C{^1^H}, and ^29^Si{^1^H} spectra were recorded using a Bruker 400 spectrometer operating at 400.1, 125.8, and 79.5 MHz, respectively; chemical shifts are quoted in ppm and are relative to tetramethylsilane (^1^H, ^13^C, and ^29^Si). Due to the low concentration of **4Th** at 298 K, ^13^C NMR chemical shifts were measured using HMBC rather than direct acquisition; HMBC overcomes the low sensitivity of ^13^C{^1^H} NMR spectroscopy for collecting the slow-relaxing, low intensity signal for carbene by transferring magnetization from the coupled ^1^H nuclei. Samples were prepared in the glove box and placed in J. Young PTFE 5 mm screw-topped borosilicate NMR tubes. FTIR spectra were recorded using a Bruker Alpha spectrometer with a Platinum-ATR module in the glove box. UV/vis/NIR spectra were recorded using a PerkinElmer Lambda 750 spectrometer where data were collected in 1 mm path length cuvettes and were run versus the appropriate reference solvent. Variable-temperature magnetic moment data were recorded in an applied dc field of 0.1 T with a Quantum Design MPMS XL7 superconducting quantum interference device magnetometer using doubly recrystallized powdered samples. Samples were carefully checked for purity and data reproducibility between several independently prepared batches for each compound examined. Care was taken to ensure complete thermalization of the sample before each data point was measured, and samples were immobilized in an eicosane matrix to prevent sample reorientation during measurements. Diamagnetic corrections were applied using tabulated Pascal constants, and measurements were corrected for the effect of the blank sample holders (flame-sealed Wilmad NMR tube and straw) and eicosane matrix. Elemental microanalyses were carried out by Mr. Martin Jennings at the Microanalytical Laboratory, Department of Chemistry, University of Manchester. Note that uranium is a weakly radioactive (α-emitter) element and should be handled with care.

The compounds [An{N(SiMe_3_)_2_}_2_(CH_2_SiMe_2_NSiMe_3_)], 1,3-dimethyl-2-methylene imidazoline (H_2_C=C(NMeCH)_2_), and [AnCl{N(SiMe_3_)_2_}_3_] were synthesized according to published procedures.^15d,29,30^

### NMR Data for Trimethylimidazolium Chloride (**5**)

^1^H NMR ((CD_3_)_2_SO, 298 K): 7.62 (s, 3H, C(CH_3_)), 3.77 (s, 6H, N(CH_3_)), 2.57 (s, 2H, C=C(H)), ppm.

### NMR Data for [U{N(SiMe_3_)_2_}_2_(CH_2_SiMe_2_NSiMe_3_)] (**6U**)

^1^H NMR (C_6_D_6_, 298 K): 11.50 (br, s, 6H, Si(CH_3_)_2_), 9.83 (br, s, 9H, Si(CH_3_)_3_CH_2_), −13.32 (br, s, 36H, Si(CH_3_)_3_), −118.59 (br, s, 2H, U–C(H_2_)) ppm.

### NMR Data for [Th{N(SiMe_3_)_2_}_2_(CH_2_SiMe_2_NSiMe_3_)] (**6Th**)

^1^H NMR (C_6_D_6_, 298 K): 0.93 (br, s, 2H, Th–C(H_2_)), 0.54 (br, s, 6H, Si(CH_3_)_2_), 0.36 (br, s, 36H, Si(CH_3_)_3_), 0.34 (br, s, 9H, Si(CH_3_)_3_CH_2_) ppm. ^29^Si{^1^H} NMR (C_6_D_6_, 298 K): δ −11.21, −12.13, −32.87 ppm. ^13^C{^1^H} NMR (C_6_D_6_, 298 K): 68.36 (SiCH_2_), 5.70 (Si(CH_3_)_2_), 4.56 (Si(CH_3_)_3_), 3.46 (Si(CH_3_)_3_) ppm.

### Preparation of [U{N(SiMe_3_)_2_}_2_(CH_2_SiMe_2_NSiMe_3_)(MIC)] (**4U**)

Method A involving [U(Cl){N(SiMe_3_)_2_}_3_] (**2U**): To a cold (−20 °C) solution of **2U** (0.50 g, 0.65 mmol) in toluene (40 mL) was added dropwise a solution of **3** (0.15 g, 1.36 mmol) in toluene (10 mL) for over 5 min with stirring. The formation of trimethylimidazolium chloride as an off-white precipitate was observed immediately. The reaction mixture was then stirred for 72 h at room temperature. The resultant dark yellow mixture was subsequently filtered through a celite-packed coarse porosity frit to obtain a dark brown filtrate, which was concentrated to approximately 10 mL and stored at 2 °C for 72 h to afford **4U** as brown block crystals. Crystalline yield: 0.16 g, 39%. Anal. calcd for C_24_H_63_N_5_Si_6_U: C, 34.80; H, 7.67; and N, 8.45%. Found: C, 34.43; H, 7.73; and N, 8.59%. ^1^H NMR (C_6_D_6_, 298 K): δ 20.25 (br, s, 3H, C(CH_3_)), 11.28 (br, s, 6H, Si(CH_3_)_3_), 9.69 (br, s, 9H, Si(CH_3_)_2_), −0.99 (s, 3H, N(CH_3_)), −4.02 (br, s, 3H, N(CH_3_)), −13.04 (br, s, 36H, Si(CH_3_)_3_), −18.53 (br, s, 1H, C=C(H)), −119.84 (br, s, 2H, U–C(H_2_)) ppm. ATR-IR ν/cm^–1^: 2944 (m), 2894 (w), 1239 (s), 1183 (w), 1130 (w), 943 (s), 892 (w), 861 (w), 820 (s), 770 (m), 748 (m), 690 (w), 660 (s), 636 (w), 607 (s), 491 (w), 419 (w). Magnetic moment (SQUID, solid + eicosane): μ_eff_ (300 K) = 2.68 μ_B_ and μ_eff_ (2 K) = 0.63 μ_B_. Method B involving [U{N(SiMe_3_)_2_}_2_(CH_2_SiMe_2_NSiMe_3_)] (**6U**): To a solution of **6U** (0.05 g, 0.07 mmol) in C_6_D_6_ (0.5 mL) was added dropwise a solution of **3** (0.011 g, 0.1 mmol) in C_6_D_6_ with vigorous shaking. Inspection of the reaction mixture by ^1^H NMR spectroscopy shows the formation of **4U**.

### Preparation of [Th{N(SiMe_3_)_2_}_2_(CH_2_SiMe_2_NSiMe_3_)(MIC)] (**4Th**)

Method A involving [Th(Cl){N(SiMe_3_)_2_}_3_] (**2Th**): To a cold (−78 °C) solution of **2Th** (0.49 g, 0.66 mmol) in toluene (40 mL) was added dropwise a solution of **3** (0.15 g, 1.36 mmol) in toluene (10 mL) for over 5 min. The formation of trimethylimidazolium chloride as an off-white precipitate was observed immediately. The reaction mixture was then stirred for 72 h at room temperature. The resultant dark yellow mixture was subsequently filtered through a celite-packed coarse porosity frit to obtain a bright yellow, clear filtrate, which was concentrated to approximately 10 mL and stored at 2 °C for 72 h to afford **4Th** as colorless crystals. Crystalline yield: 0.194 g, 36%. Anal. calcd for C_24_H_63_N_5_Si_6_Th: C, 35.05; H, 7.72; and N, 8.52%. Found: C, 34.82; H, 7.74; and N, 8.36%. In solution, **4Th** is in equilibrium with [Th{N(SiMe_3_)_2_}_2_(CH_2_SiMe_2_NSiMe_3_)] (**6Th**) and **3**. ^1^H NMR (C_6_D_6_, 298 K): δ 6.48 (s, 1H, C=C(H)), 5.45 (s, 3H, C(CH_2_) **3**), 3.46 (s, 3H, N(CH_3_)), 2.59 (s, 6H, N(CH_3_) **3**), 2.56 (s, 2H, C=C(H) **3**), 2.16 (s, 3H, N(CH_3_)), 1.06 (s, 3H, C(CH_3_)), 0.80 (br, s, 2H, Th–C(H_2_), **6Th**), 0.73 (br, s, 6H, Si(CH_3_)_2_), 0.66 (br, s, 2H, Th–C(H_2_)), 0.57 (br, s, 6H, Si(CH_3_)_2_, **6Th**), 0.50 (br, s, 36H, Si(CH_3_)_3_), 0.39 (br, s, 36H, Si(CH_3_)_3_, **6Th**), 0.38 (br, s, 9H, Si(CH_3_)_3_CH_2_, **6Th**), 0.34 (br, s, 9H, Si(CH_3_)_3_CH_2_) ppm. ^13^C{^1^H} NMR (C_6_D_6_, 298 K): δ 208.4 (s, C=C_carbene_), 140.1 (s, C–CH_3_), 128.9 (s, C=CH), 38.1 (s, C_carbene_N–CH_3_), 32.3 (s, HCNCH_3_), 7.7 (s, C–CH_3_), 6.4 (s, Th–CH_2_), 4.5 (m, Si(CH_3_)_3_) ppm. ^29^Si{^1^H} NMR (C_6_D_6_, 298 K): δ −10.58, −11.03 (**6Th**), −11.32, −11.41 (**6Th**), −23.96, −29.24 (**6Th**) ppm. ATR-IR ν/cm^–1^: 2959 (s), 2897 (m), 1941 (m), 1909 (m), 1540 (w), 1473 (w), 1418 (w), 1245 (s), 1090 (m), 1017 (s), 921 (s), 796 (s), 770 (w), 661 (s), 606 (s), 546 (w), 521 (w). Method B involving [Th{N(SiMe_3_)_2_}_2_(CH_2_SiMe_2_NSiMe_3_)] (**6Th**): To a solution of [Th{N(SiMe_3_)_2_}_2_(CH_2_SiMe_2_NSiMe_3_)] (0.04 g, 0.056 mmol) in C_6_D_6_ (0.5 mL) was added dropwise a solution of **3** (0.007 g, 0.064 mmol) in C_6_D_6_ with vigorous shaking. There was an immediate color change from colorless to bright yellow. Inspection of the reaction mixture by ^1^H and ^29^Si{^1^H} NMR spectroscopies shows the formation of **4Th**.

### General Computational Details

Unrestricted and restricted geometry optimizations were performed as appropriate for the full models of **4U** and **4Th** using coordinates derived from their X-ray crystal structures. No constraints were imposed on the structures during the geometry optimizations. The calculations were performed using the Amsterdam density functional (ADF) suite versions 2012.01 (geometry optimizations of full compounds, molecular orbital analysis, bond orders, and NBO analysis) and 2017 (analytical frequency and EDA-NOCV calculations).^31,32^ The DFT geometry optimizations employed Slater-type orbital (STO) triple-ζ-plus polarization all-electron basis sets (from the ZORA/TZP database of the ADF suite). Scalar relativistic approaches were used within the ZORA Hamiltonian^33−35^ for the inclusion of relativistic effects and the local density approximation with the correlation potential was used in all the calculations based on the study by Vosko et al.^36^ Gradient corrections were performed using the functionals of Becke and Perdew.^37,38^ MOLEKEL^39^ was used to prepare the three-dimensional plots of the electron density. NBO analyses were carried out with NBO 6.0.^40^ The atoms-in-molecules analysis^41,42^ was carried out with Xaim-1.0.^43^

### Energy Decomposition Analysis (EDA)

EDA (also known as extended transition-state method, ETS) was developed independently by Morokuma^44^ and Ziegler and Rauk.^45^ It analyses the interaction energy, Δ*E*_int_, of a bond in the molecule A–B with fragments A and B in the frozen geometry of AB and the particular electronic reference state. The interaction energy can be described as the sum of three interactions:
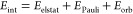
Δ*E*_elstat_ describes the quasi-classical Coulomb interaction between the unperturbed charge distributions of the fragments A and B. Δ*E*_Pauli_ is the Pauli repulsion, which is destabilizing and describes the interaction between electrons of the same spin between the two fragments. The third interaction Δ*E*_orb_ is the orbital interaction, which includes the charge transfer and polarization effects. Further details on the EDA method and examples on bond analysis using EDA can be found in the literature.

An extension to the EDA scheme, which was used in this study, is EDA-NOCV. It combines EDA with the decomposition of NOCV.^46^ Thereby, pairwise energy contributions for each pair of interacting orbitals are provided and Δ*E*_orb_ can be analyzed by single orbital contributions:
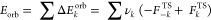
where −*F*_–*k*_^TS^ and *F*_*k*_^TS^ are the diagonal transition-state Kohn–Sham matrix elements that correspond to the NOCVs with eigenvalues −ν_*k*_ and ν_*k*_. This decomposition scheme allows for the interpretation of bonding interactions in molecules without symmetry as the deformation density is also based on the NOCVs and can be plotted to visualize the single contributions. Additionally, Δ*E*_*k*_^orb^ provides quantitative interpretation.

## Supporting Information Available

Experimental and computational details, NMR spectra, infrared spectra, optical spectra, structure of **4Th**, magnetic plots, van’t Hoff analysis data, and computational data (PDF)
Crystallographic data for **4Th** (XYZ)
Crystallographic data for **4U** (XYZ)

## Supplementary Material

om2c00120_si_001.pdf

om2c00120_si_002.xyz

om2c00120_si_003.xyz

## Accession Codes

CCDC 1915357–1915358 contain the supplementary crystallographic data for this paper. These data can be obtained free of charge via www.ccdc.cam.ac.uk/data_request/cif, or by emailing data_request@ccdc.cam.ac.uk, or by contacting The Cambridge Crystallographic Data Centre, 12 Union Road, Cambridge CB2 1EZ, UK; fax: +44 1223 336033.

CCDC 1915357 and 1915358 contain the supplementary crystallographic data for this study. These data can be obtained free of charge via www.ccdc.cam.ac.uk/data_request/cif, or by emailing data_request@ccdc.cam.ac.uk, or by contacting the Cambridge Crystallographic Data Centre, 12 Union Road, Cambridge, CB2 1EZ, UK; fax: +44 1223 336033.

## Dedication

Dedicated to Professor Glen Deacon to mark the occasion of his 85th birthday.
