# Supplementary material for: Mesoionic Carbene Complexes of Uranium(IV) and Thorium(IV)
Source: Organometallics. 2022 May 18;41(11):1353–63. doi: 10.1021/acs.organomet.2c00120 (PMC9490841; doi:10.1021/acs.organomet.2c00120)
Supplement: Supplementary file 1 — om2c00120_si_001.pdf [file om2c00120_si_001.pdf]

– Supporting Information –

Mesoionic Carbene Complexes of Uranium(IV) and Thorium(IV)

John A. Seed, Lisa Vondung, Ralph W. Adams, Ashley J. Wooles, Erli Lu, Stephen T. Liddle*

Department of Chemistry, The University of Manchester, Oxford Road, Manchester, M13 9PL, UK.

*Correspondence email: steve.liddle@manchester.ac.uk

General note

Trace impurities are often observed in NMR spectra of organoactinide complexes. Some resonances cannot be assigned, but common occurrences are the residual protio resonance of benzene (7.16 ppm), $HN(SiMe_3)_2$ (0.16 ppm) and poly(dimethylsiloxane) (0.29 ppm).[1] These are not annotated on the NMR spectra below for clarity.

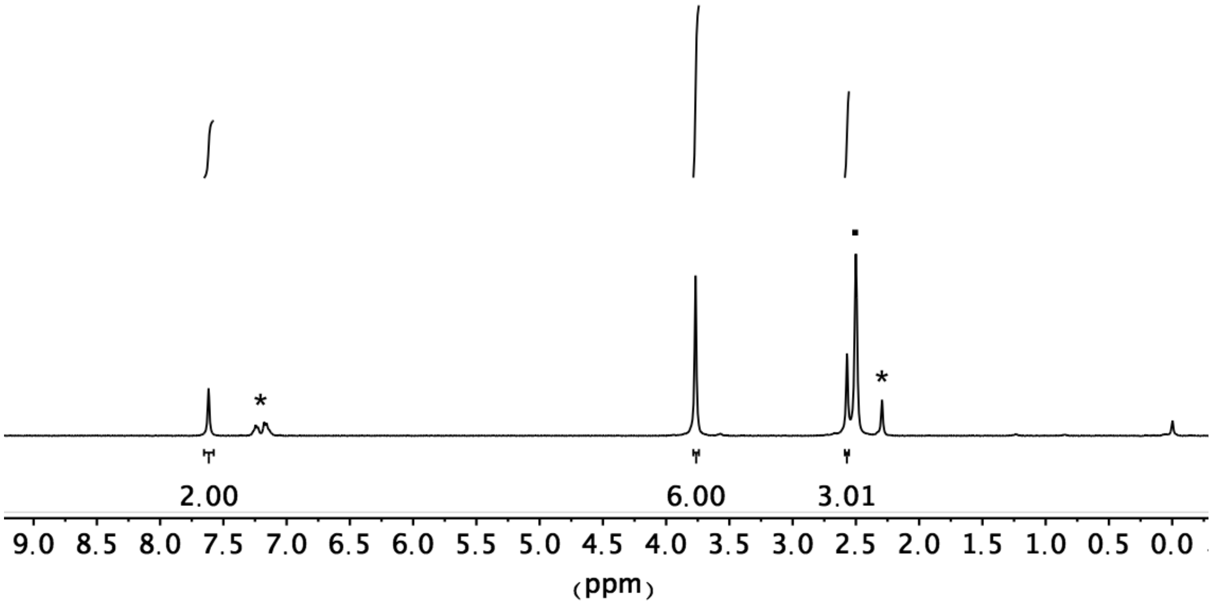

Figure S1. ^1H NMR spectrum of **5** in $(CD_3)_2SO$ – asterisk (*) denotes trace impurities; dot (•) denotes $(CD_3)_2SO$.

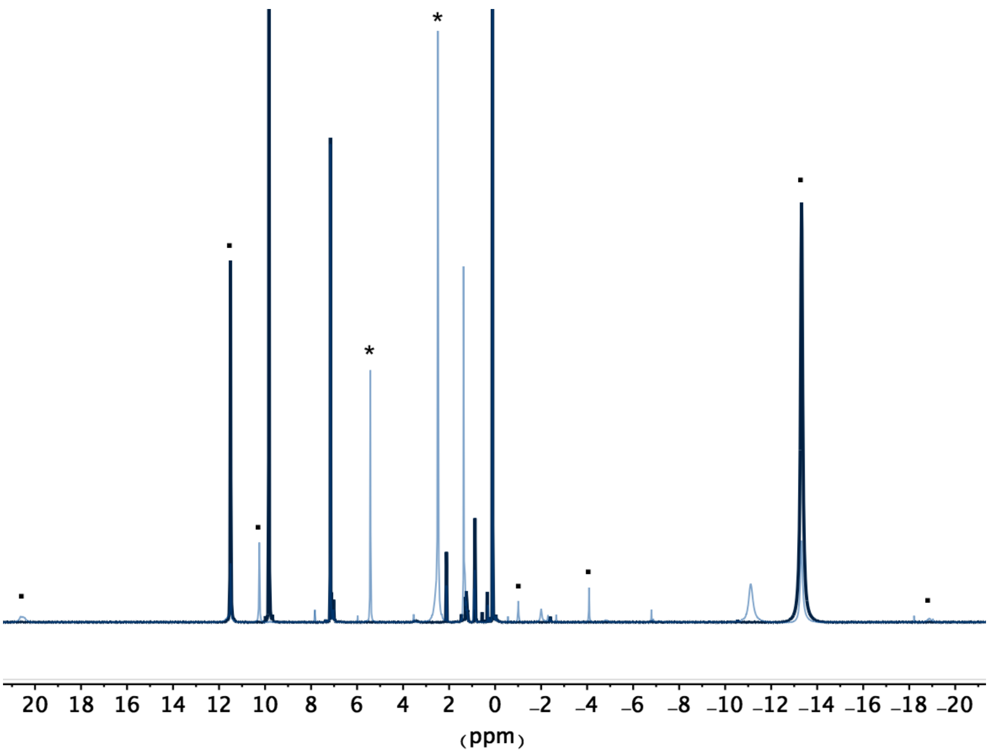

Figure S2. Superimposed ^{1}H NMR spectra of [U{N(SiMe$_3$)$_2$}$_2$(CH$_2$SiMe$_2$NSiMe$_3$)] before the addition of **3** (black) and after (blue) in C_6D_6 – asterisk (*) denotes **3**; dot (•) denotes **4U**.

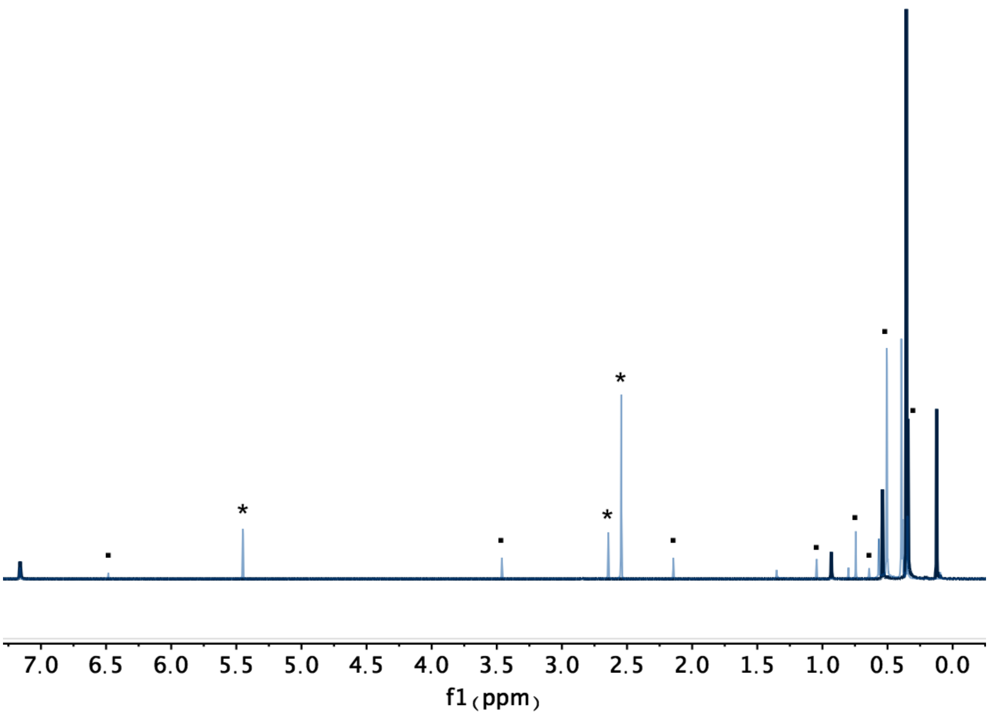

Figure S3. Stacked ^{1}H NMR spectra of [Th{N(SiMe$_3$)$_2$}$_2$(CH$_2$SiMe$_2$NSiMe$_3$)] before the addition of **3** (black) and after (blue) in C_6D_6 – asterisk (*) denotes **3**; dot (•) denotes **4Th**.

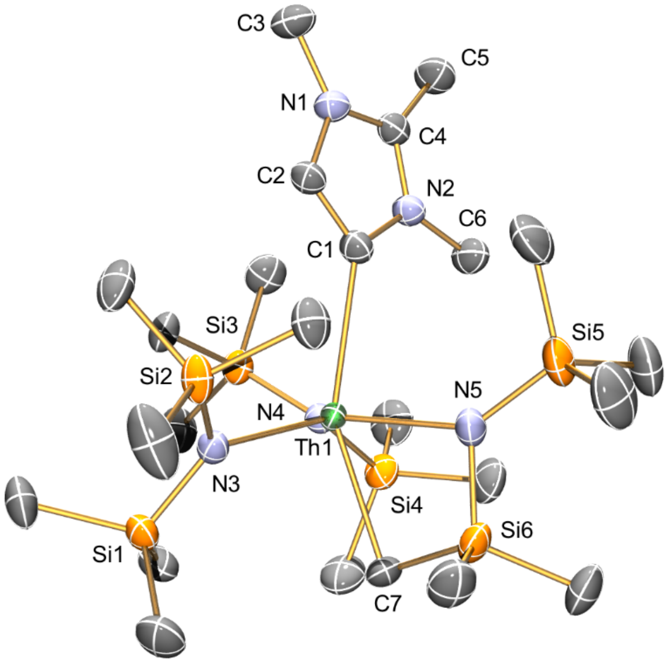

Figure S4. Molecular structure of **4Th** at 150 K. Displacement ellipsoids are set at 30% with hydrogens omitted for clarity.

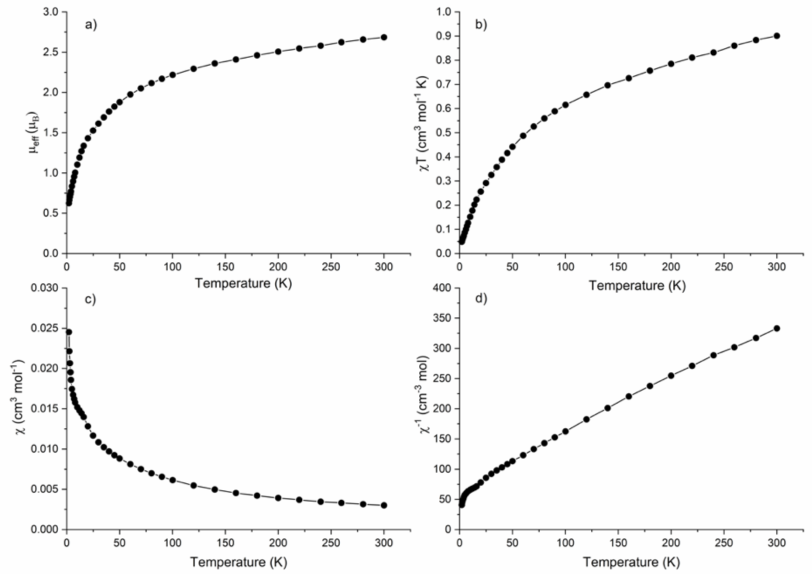

Figure S5. Variable-temperature SQUID data of a powdered sample of **4U.** Lines are a guide to the eye only: a) μ_{eff} vs T; b) χT vs T; c) χ vs T; d) χ^{-1} vs T.

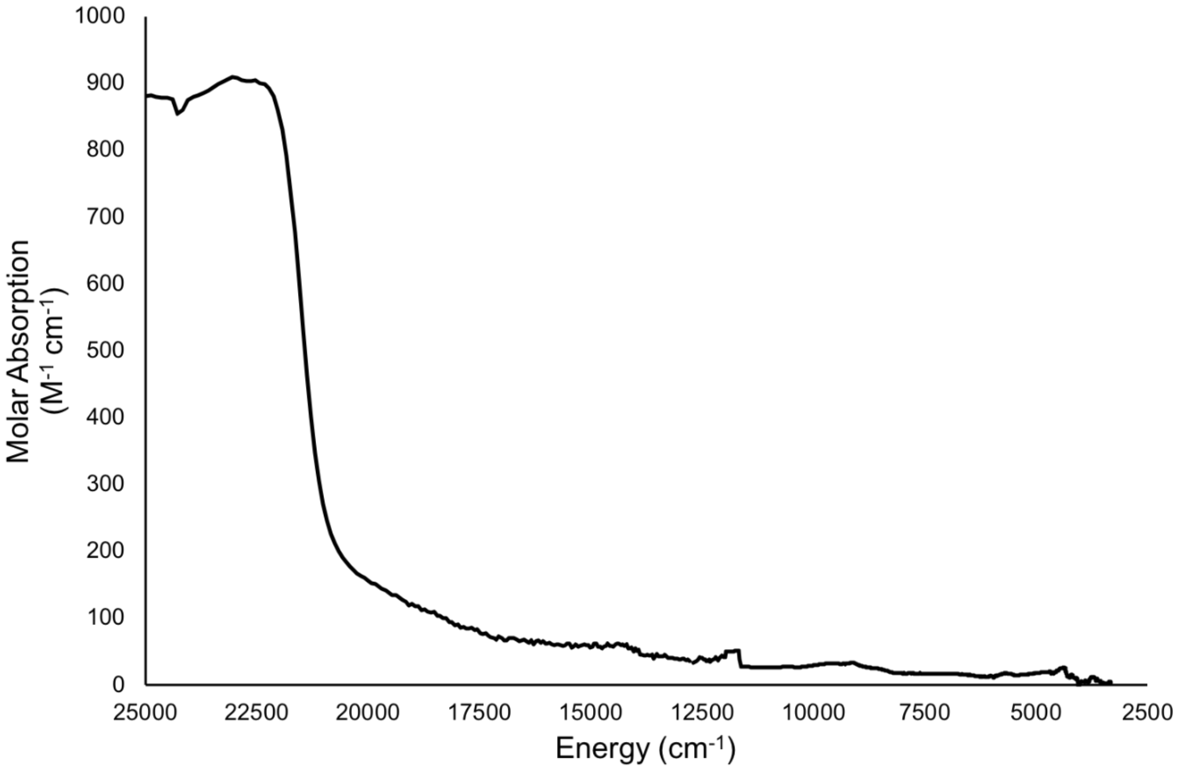

Figure S6. UV/Vis/NIR spectrum of **4U** in toluene over the range 2,500-25,000 cm^{-1}.

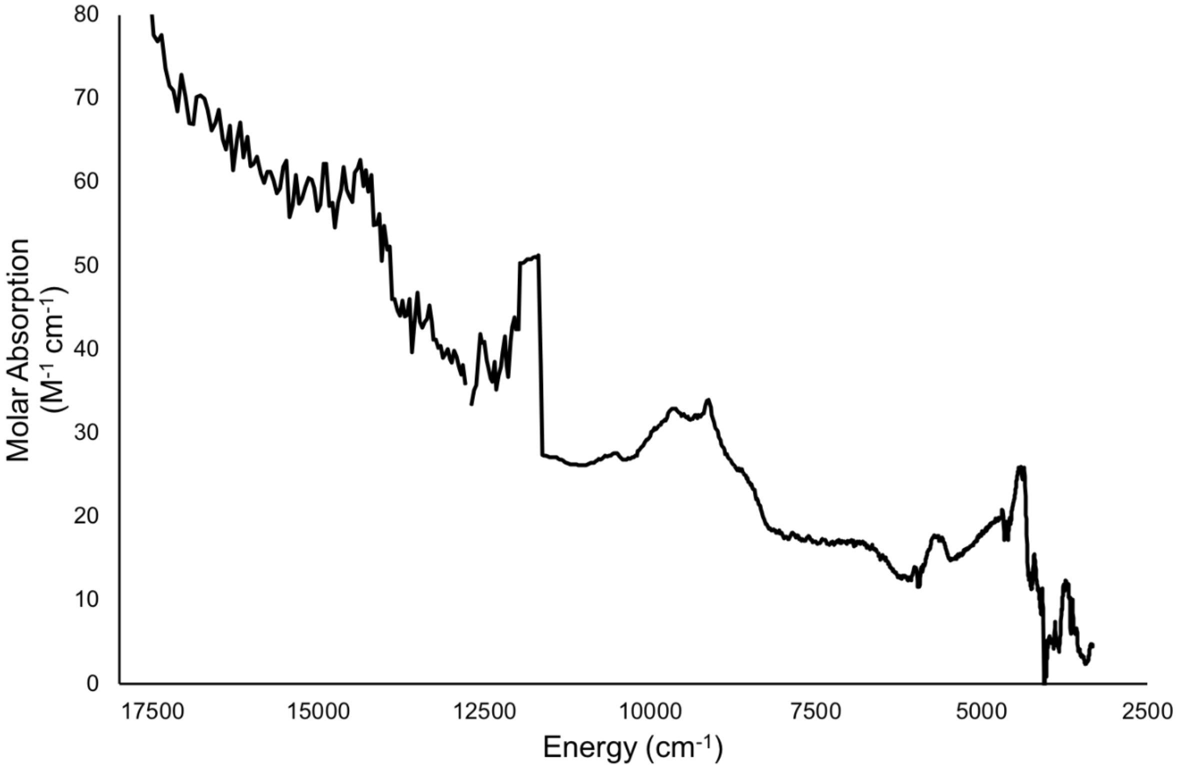

Figure S7. UV/Vis/NIR spectrum of **4U** in toluene zoomed-in over the range 3,000-18,000 cm^{-1}.

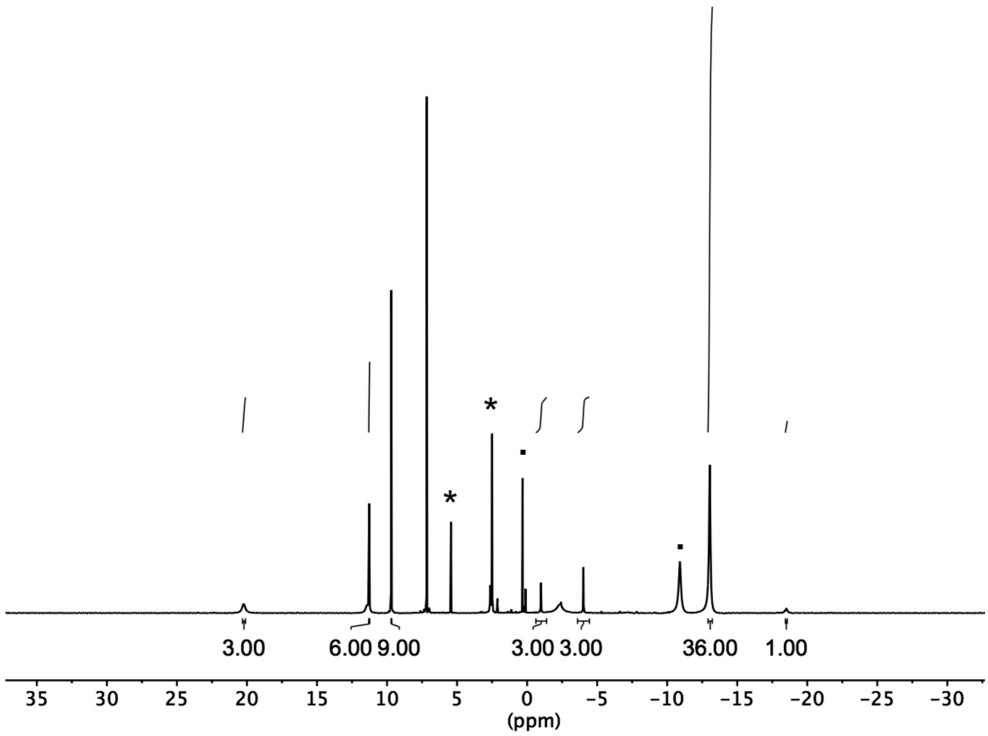

Figure S8. ^{1}H NMR spectrum of **4U** in C_6D_6 – asterisk (*) denotes trace **3**; dot (•) denotes trace impurities.

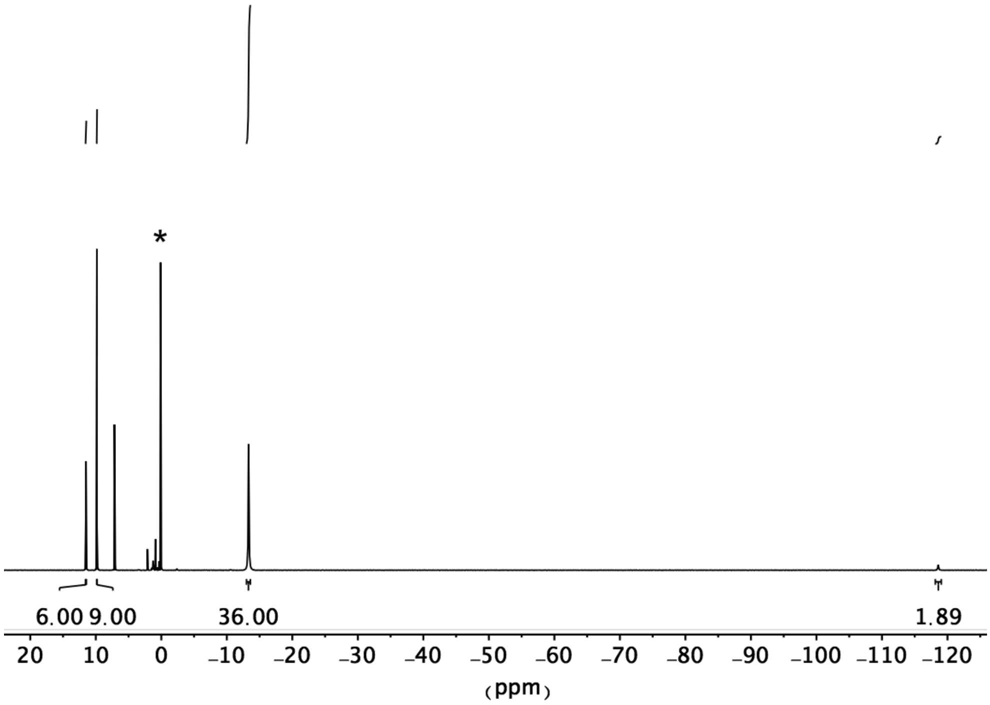

Figure S9. ^{1}H NMR spectrum of $[U\{N(SiMe_3)_2\}_2(CH_2SiMe_2NSiMe_3)]$ in C_6D_6 – asterisk (*) denotes trace $HN(SiMe_3)_2$

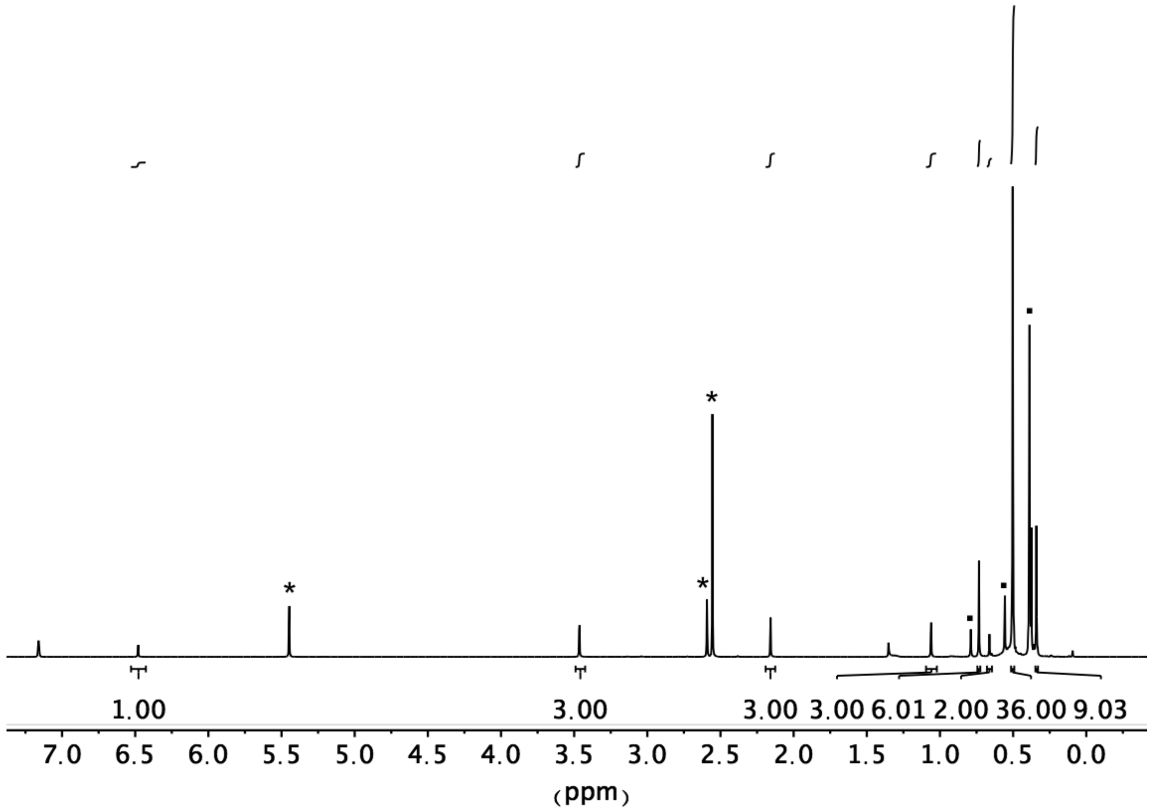

Figure S10. ^{1}H NMR spectrum of **4Th** in C_6D_6 – asterisk (*) denotes **3**; dot (•) denotes $[Th\{N(SiMe_3)_2\}_2(CH_2SiMe_2NSiMe_3)]$.

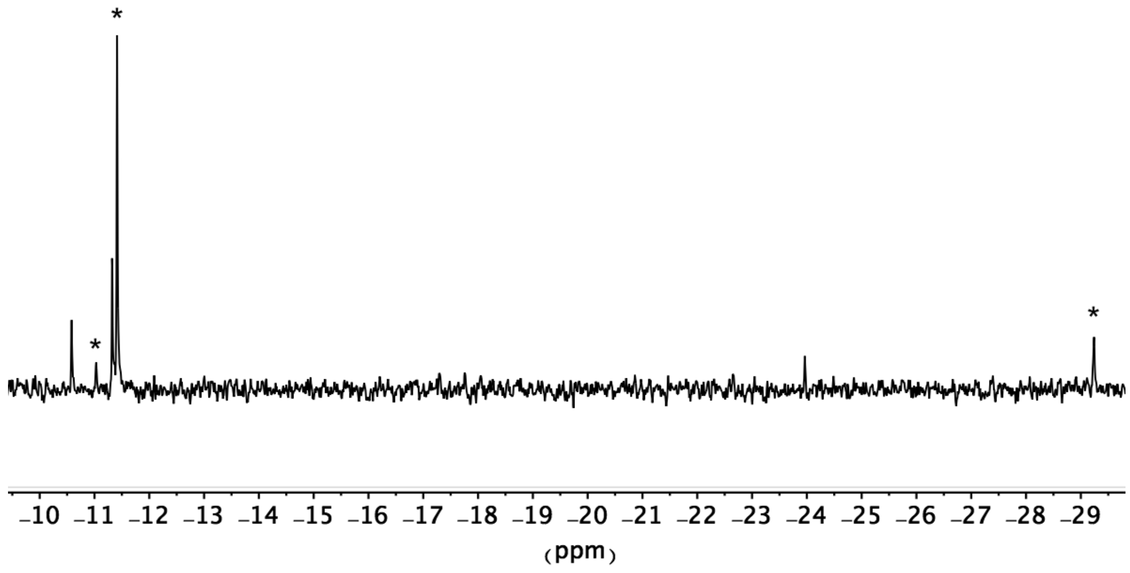

Figure S11. $^{29}Si\{^{1}H\}$ NMR spectrum of **4Th** in C_6D_6 – asterisk (*) denotes $[Th\{N(SiMe_3)_2\}_2(CH_2SiMe_2NSiMe_3)]$.

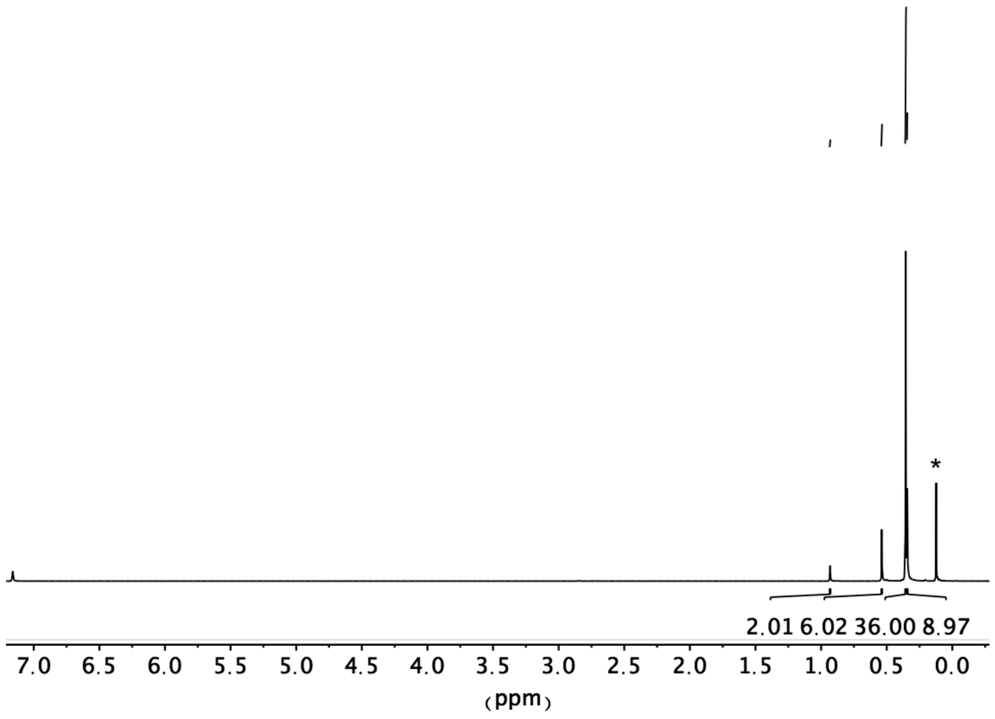

Figure S12. ^{1}H NMR spectrum of [Th{N(SiMe$_3$)$_2$}$_2$(CH$_2$SiMe$_2$NSiMe$_3$)] in C_6D_6 – asterisk (*) denotes trace HN(SiMe$_3$)$_2$

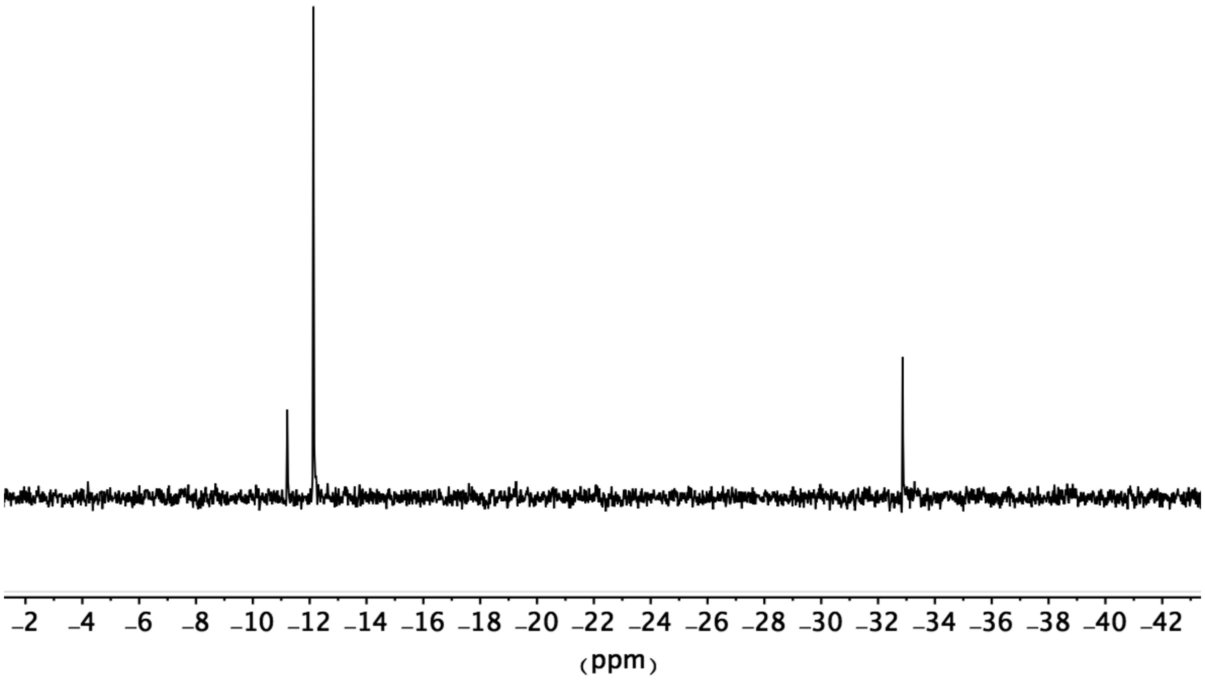

Figure S13. $^{29}Si\{^{1}H\}$ NMR spectrum of [Th{N(SiMe$_3$)$_2$}$_2$(CH$_2$SiMe$_2$NSiMe$_3$)] in C_6D_6.

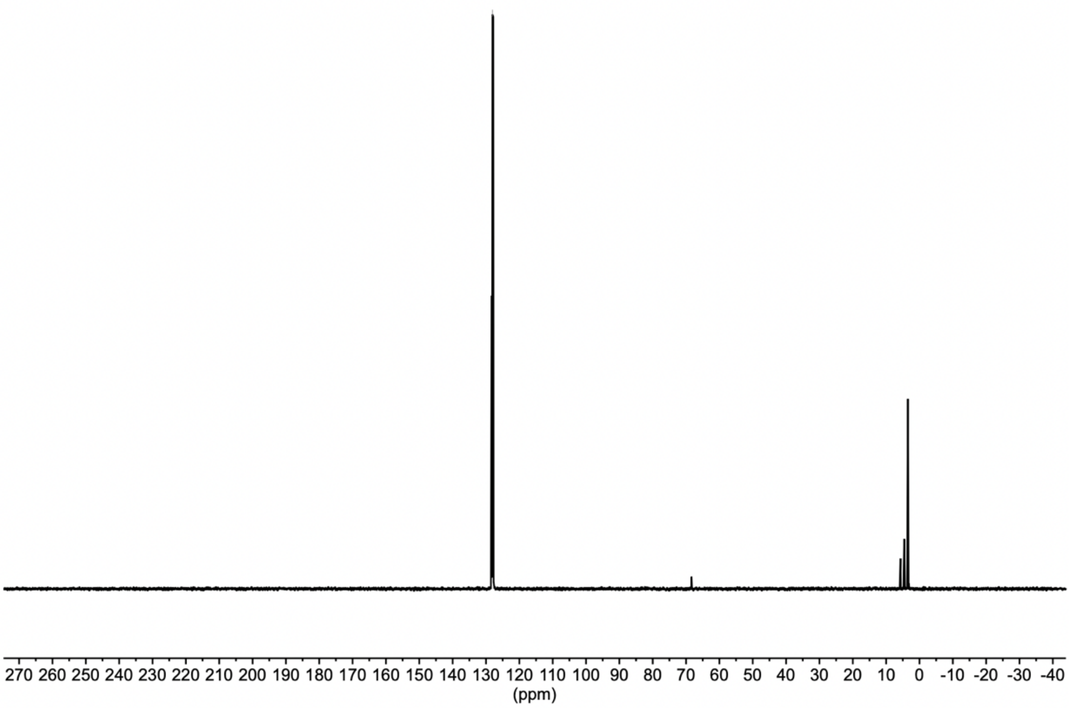

Figure S14. $^{13}C\{^{1}H\}$ NMR spectrum of [Th{N(SiMe$_3$)$_2$}$_2$(CH$_2$SiMe$_2$NSiMe$_3$)] in C_6D_6.

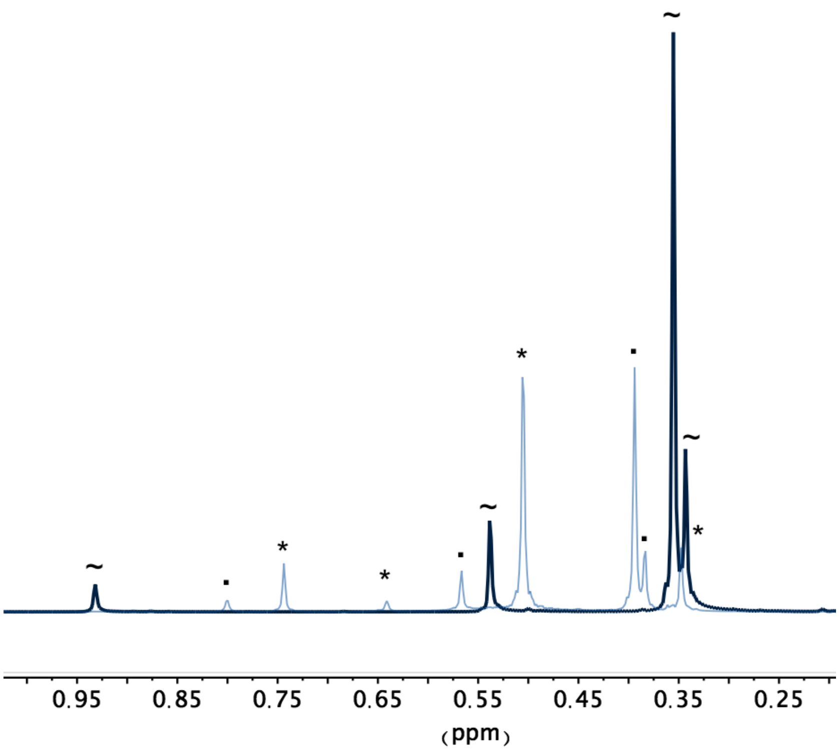

Figure S15. Superimposed ^{1}H NMR spectra of [Th{N(SiMe$_3$)$_2$}$_2$(CH$_2$SiMe$_2$NSiMe$_3$)] (black) and crystalline **4Th** (blue) in C_6D_6 zoomed in between 1.0 and 0 ppm – tilde (~) denotes pure [Th{N(SiMe$_3$)$_2$}$_2$(CH$_2$SiMe$_2$NSiMe$_3$)]; dot (•) denotes shifted resonances of [Th{N(SiMe$_3$)$_2$}$_2$(CH$_2$SiMe$_2$NSiMe$_3$)]; asterisk (*) denotes **4Th**.

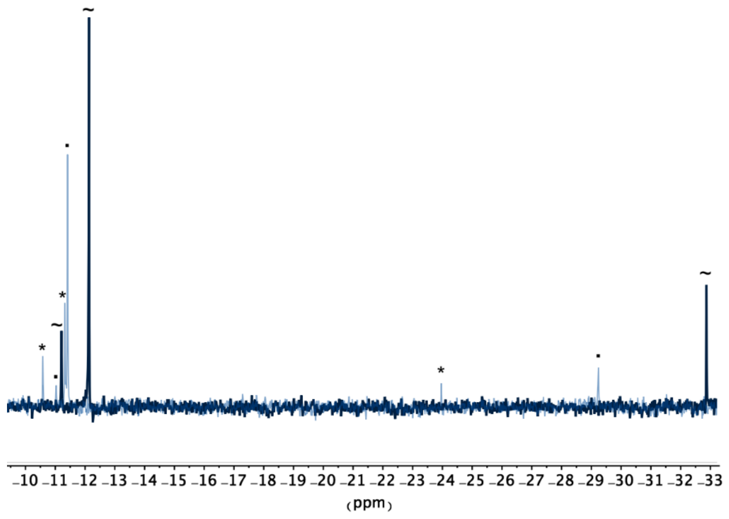

Figure S16. Superimposed $^{29}Si\{^1H\}$ NMR spectra of $[Th\{N(SiMe_3)_2\}_2(CH_2SiMe_2NSiMe_3)]$ (black) and crystalline **4Th** (blue) in C_6D_6 – tilde (~) denotes pure $[Th\{N(SiMe_3)_2\}_2(CH_2SiMe_2NSiMe_3)]$; dot (•) shifted resonances of $[Th\{N(SiMe_3)_2\}_2(CH_2SiMe_2NSiMe_3)]$; asterisk (*) denotes **4Th**.

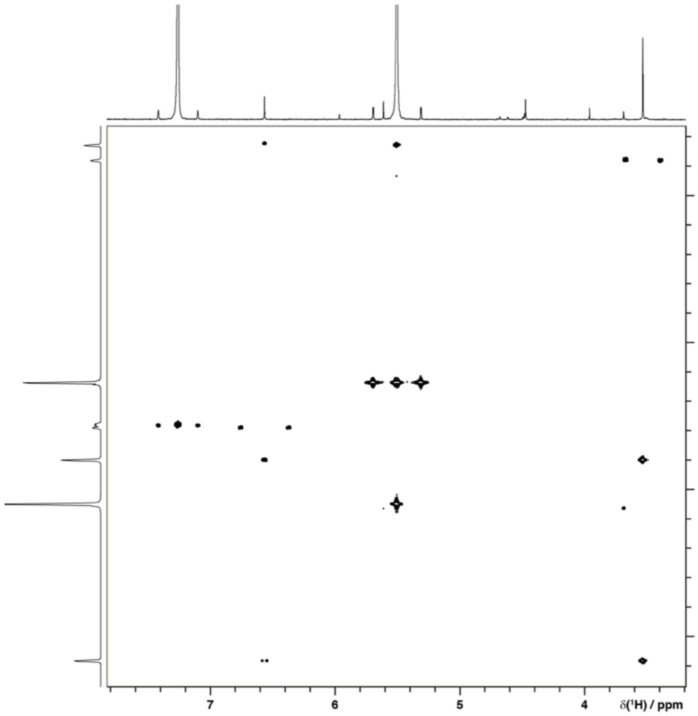

Figure S17. Selected region of ^1H-^{13}C HMBC NMR spectrum of **4Th** in C_6D_6 showing correlations to the carbene signal at 208 ppm. The displayed region of the HMBC spectrum is projected onto the ^{13}C axis to give a 1D trace, allowing extraction of the **4Th** ^{13}C chemical shifts, including C=$C_{carbene}$ and C=*C*H, in the presence of much stronger solvent and **6Th** signals. For clarity, ^{13}C signals originating from HMBC correlations to ^1H signals below 3 ppm are not shown. The ^1H axis shows the conventional 1D ^1H spectrum with the signals from residual solvent and **3** at 7.16 ppm and 5.40 ppm truncated.

Van't Hoff Analysis

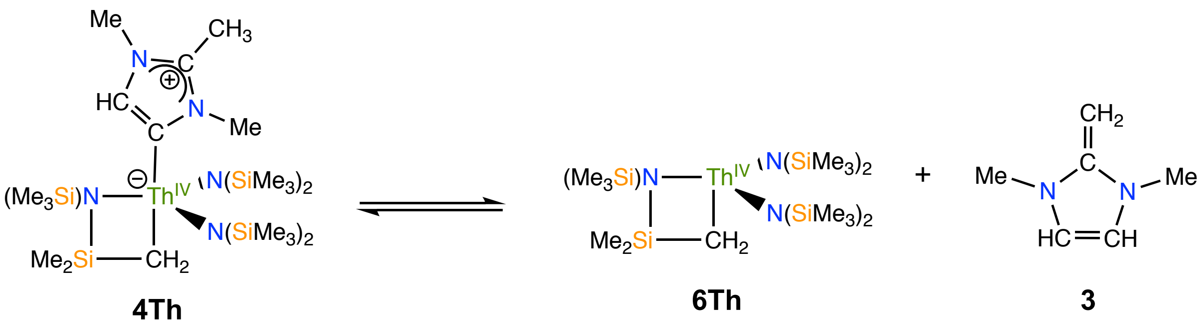

Scheme S1. Equilibrium reaction scheme for the conversion of **4Th** into **6Th** and **3**.

A Van't Hoff analysis utilising variable temperature ^{1}H NMR spectroscopy experiments, over the temperature range 298.15-253.15 K, allowed for the determination of the equilibrium constant, K_{eq}, for the conversion of **4Th** into **3** and **6Th**, Scheme S1. The procedure followed was adapted from that used by Power and colleagues.[2]

$$K_{eq} = \frac{[\mathbf{3}][\mathbf{6}\boldsymbol{Th}]}{[\mathbf{4}\boldsymbol{Th}]}$$

Equation 1

$$lnK_{eq} = -\frac{\Delta H}{RT} + \frac{\Delta S}{R}$$

Equation 2

Equation 1 allowed for the determination of the value of K_{eq} using the integrals of the {N(Si(C*H*$_3$)$_3$)$_2$} groups of **4Th** and **6Th**, Figure S18, and the N(C*H*$_3$) groups of **3**, Figure S19. Whilst the Van't Hoff equation, **Equation 2**, was used to determine ΔH and ΔS from the slope and the intercept of the plot of RlnK$_{eq}$ against 1000/T, Figure S19. The uncertainty of integration is assumed to be 10% due to line broadening.

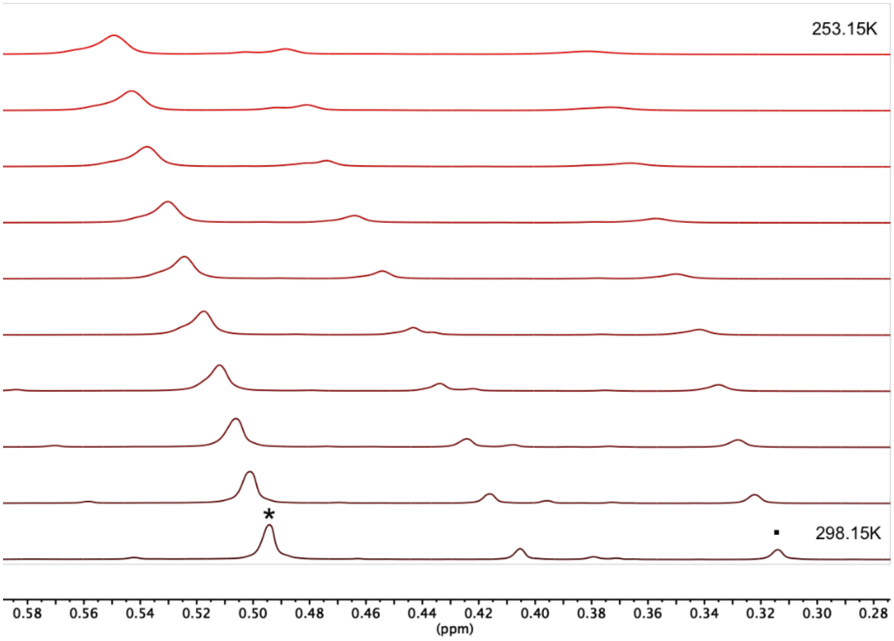

Figure S18. variable temperature NMR experiment for the conversion of **4Th** into **6Th** and **3** ranging from 298.15K (bottom) to 253.15K (top), decreasing in 5K increments. These spectra are zoomed in between ~0.58 and 0.28 ppm to highlight the resonances attributed to the {N(Si(CH_3)$_3$)$_2$} groups of **4Th** and **6Th** - asterisk (*) denotes **4Th**; dot (•) denotes **6Th**.

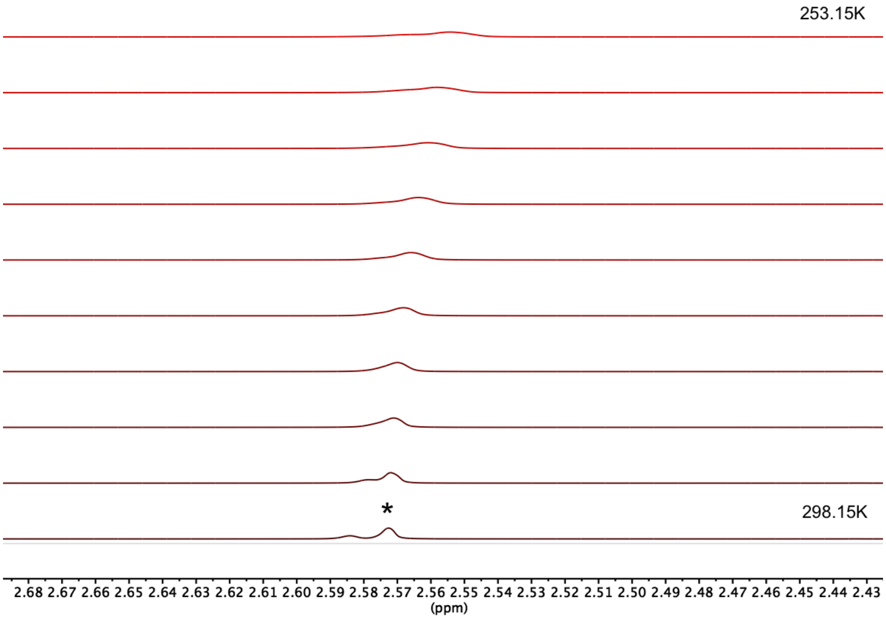

Figure S19. variable temperature NMR experiment for the conversion of **4Th** into **6Th** and **3** ranging from 298.15K (bottom) to 253.15K (top), decreasing in 5K increments. These spectra are zoomed in between ~2.68 and 2.43 ppm to highlight the resonance attributed to the N(CH_3) groups of **3** - asterisk (*) denotes **3**.

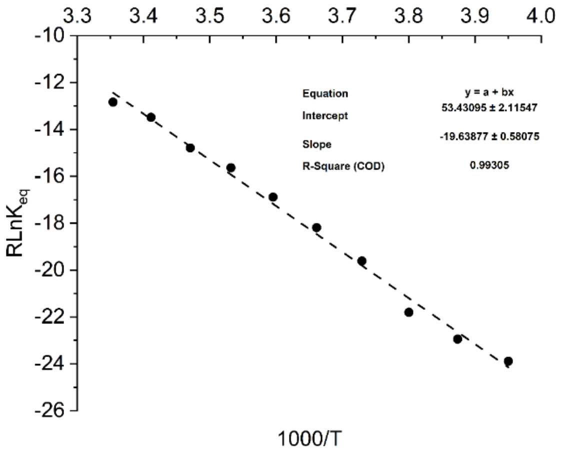

Figure S20. van't Hoff plot for the conversion of **4Th** into **6Th** and **3** in toluene-d_8: concentration: 0.0778 mol L^{-1}.

Computational Data

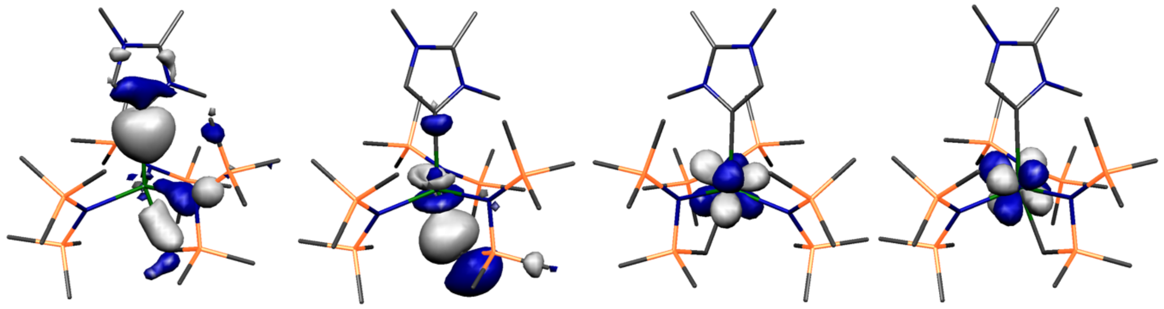

Figure S21. Frontier Kohn Sham Molecular Orbitals of **4U** with H-atoms omitted for clarity. Left to right: HOMO−16 (194a, −6.802 eV); HOMO−2 (208a, −4.315 eV); HOMO−1 (209a, −2.535 eV); HOMO (210a, −2.510 eV).

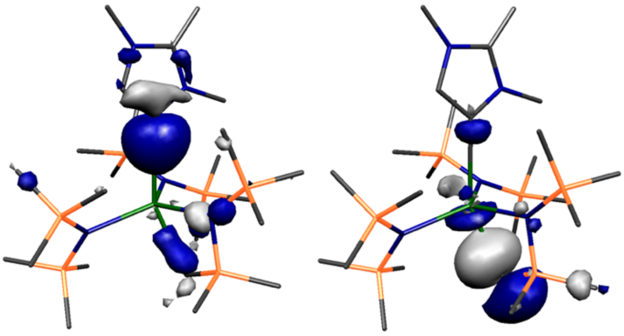

Figure S22. Frontier Kohn Sham Molecular Orbitals of **4Th** with H-atoms omitted for clarity**.** Left to right: HOMO−14 (194a, −6.727 eV); HOMO (208a, −4.413 eV).

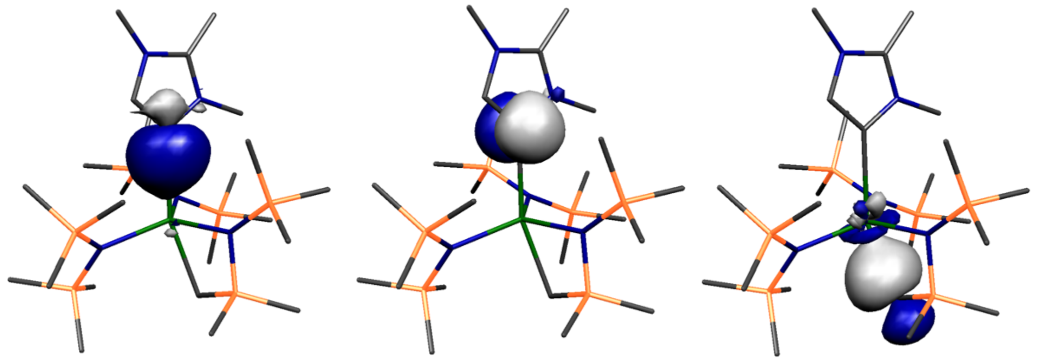

Figure S23. NBOs of **4U** with H-atoms omitted for clarity. Left to right: U-MIC σ-bond; U-MIC π-bond; U-C_{cyclo} σ-bond.

EDA-NOCV results

Table S1. EDA-NOCV results (BP86/TZ2P+) for **4U** and **4Th** from fragmentation in triplet [An{N(SiMe$_3$)$_2$}$_2$(CH$_2$SiMe$_2$NSiMe$_3$)] and the neutral singlet mesoionic carbene.

Energy terms[a]	**4U**[b]	**4Th**[b]
ΔE_{int}	–39.0	–29.9
ΔE_{Pauli}	98.9	79.4
ΔE_{elstat}	–85.3 (61.4%)	–75.6 (69.2%)
ΔE_{orb}	–52.6 (38.1%)	–33.7 (30.8%)
ΔE_{prep}	9.4	7.4
$-D_e$	–29.5	–22.5

[a] Energy contributions are given in kcal/mol.
[b] The values in parentheses gives the percentage contribution to the total attractive interactions: $\Delta E_{elstat} + \Delta E_{orb}$.

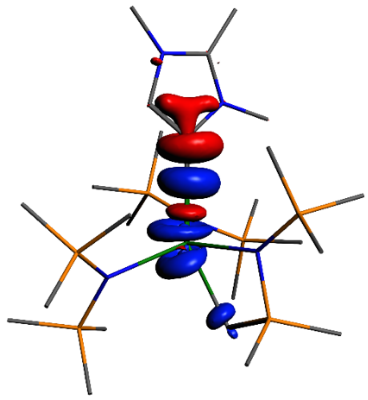

Figure S24. Deformation density with contribution >5 kcal/mol for **4Th**. Contribution to ΔE_{orb}: ΔE_1 = –19.6 kcal/mol. Eigenvalue: $|\nu_1|$= 0.45. Charge flow is from red to blue. Contour value is set to $|\Delta\rho|$=0.003 a.u. and H-atoms omitted for clarity.

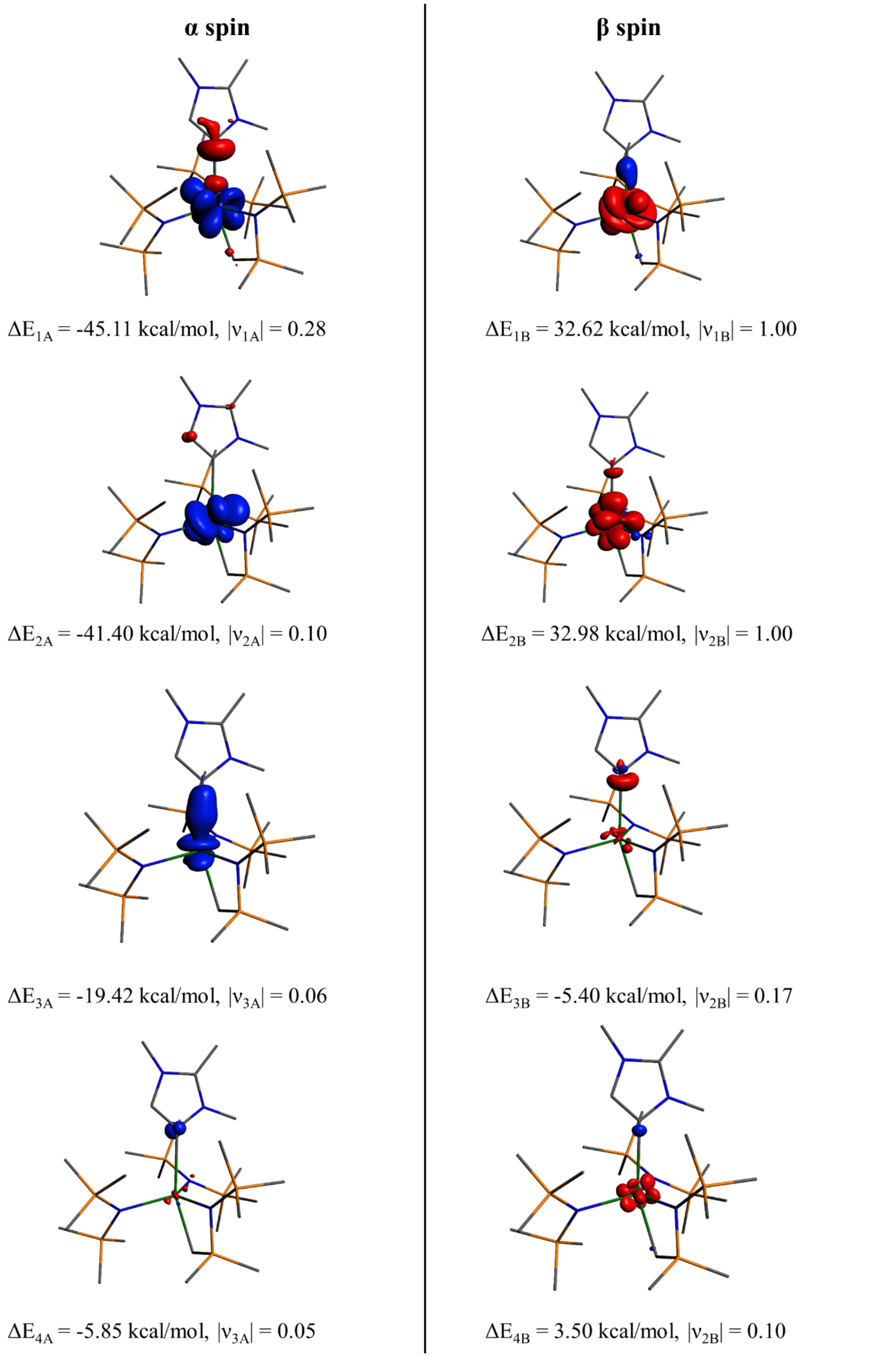

Figure S25. Deformation densities with contribution >5 kcal/mol for **4U** and corresponding energy contribution ΔE_i to ΔE_{orb} and eigenvalues $|\nu_i|$. Charge flow is from red to blue. Contour values are set to $|\Delta\rho|$=0.003 a.u. and H-atoms omitted for clarity.

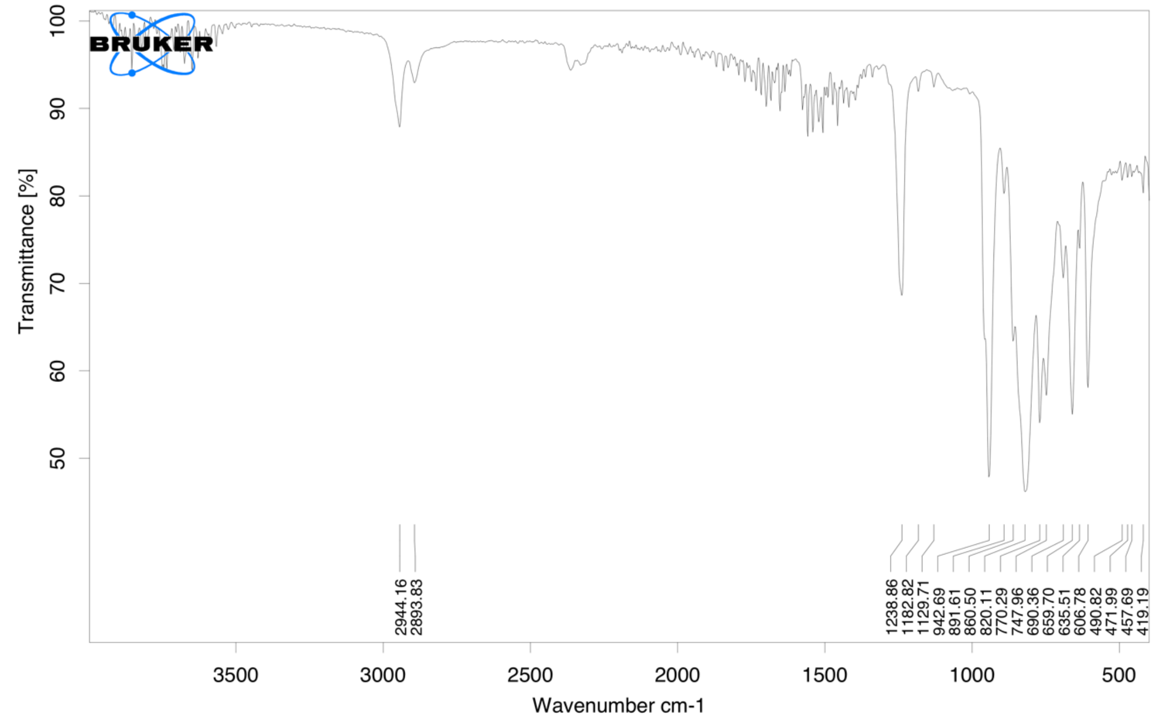

Figure S26. FTIR spectrum of **4U.**

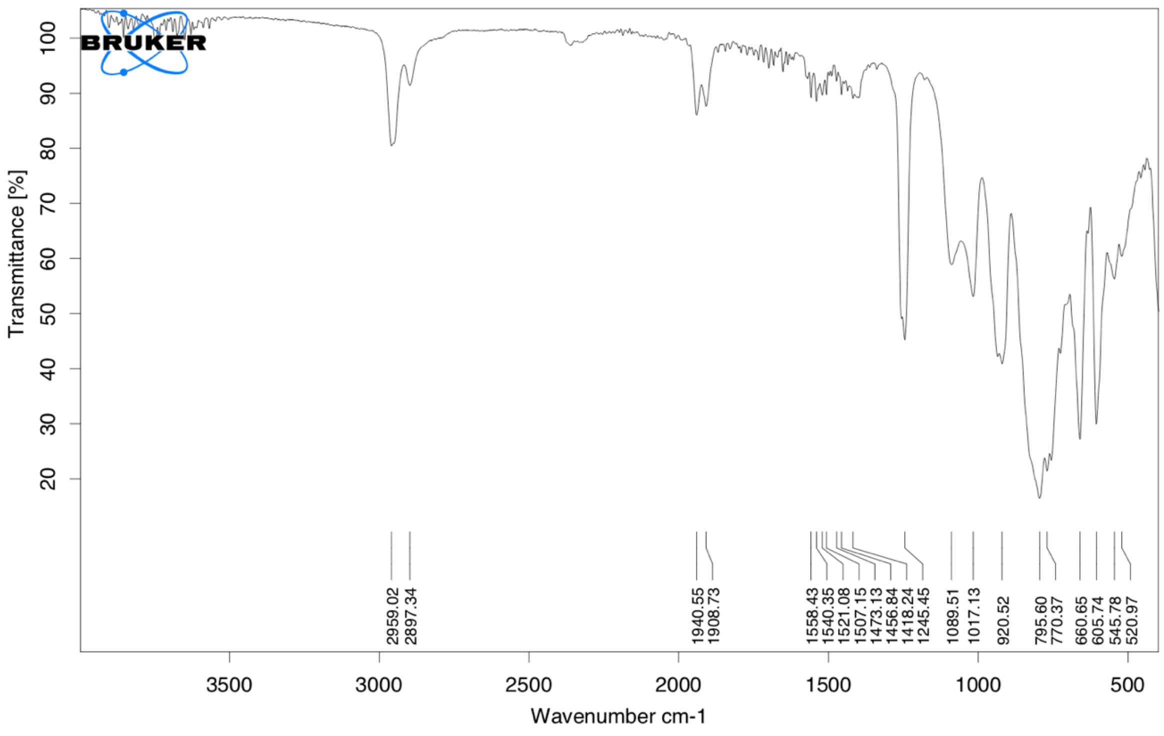

Figure S27. FTIR spectrum of **4Th.**

Table S2. Experimental X-ray crystallographic details for **4U** and **4Th**.

	**4U**	**4Th**
Formula	$C_{24}H_{63}N_5Si_6U$	$C_{31}H_{71}N_5Si_6Th$
Fw, g mol^{-1}	828.36	914.50
Cryst size, mm	0.11 x 0.065 x 0.051	0.389 x 0.322 x 0.282
Crystal system	orthorhombic	monoclinic
Space group	Pna21	I2/a
Collection Temperature (K)	150(2)	150(2)
a, (Å)	23.6670(2)	20.4840(6)
b, (Å)	12.82144(10)	22.3967(5)
c, (Å)	12.92519(11)	21.7849(6)
α, (°)	90	90
β, (°)	90	112.979(3)
γ, (°)	90	90
V, (Å^3)	3922.09(6)	9201.3(5)
Z	4	8
ρ_{calc} g cm^{-3}	1.403	1.320
μ, mm^{-1}	13.552	3.422
No. of reflections measured	14806	42667
No. of unique reflections, Rint	6374, 0.0282	11416, 0.0444
No. of reflections with $F^2 > 2s(F^2)$	6119	9112
Transmission coefficient range	0.422-0.691	0.408-1.000
R, R_w[a] ($F^2 > 2s(F^2)$)	0.0390, 0.1019	0.0281, 0.0549
R, R_w[a] (all data)	0.0400, 0.1030	0.0452, 0.0585
S[a]	1.058	1.030
Parameters, Restraints	345, 1	438, 67
Max.,min. difference map, e Å^{-3}	2.111, -3.072	1.240, -0.731

References

1. Fulmer, G. R.; Miller, A. J. M.; Sherden, N. H.; Gottlieb, H. E.; Nudelman, A.; Stoltz, B. M.; Bercaw, J. E.; Goldberg, K. I. NMR Chemical Shifts of Trace Impurities: Common Laboratory Solvents, Organics, and Gases in Deuterated Solvents Relevant to the Organometallic Chemist. *Organometallics* **2010**, *29*, 2176-2179.

2. Gullet, K. L.; Lai, T. Y.; Chen, C.-Y.; Fettinger, J. C.; Power, P. P. Reversible Binding of Ethylene and Propylene by Germylenes. *Organometallics* **2019**, *38*, 1425-1428.
